# HNF4α-HKDC1 axis orchestrates a metabolic rewiring to promote migration and metastasis in advanced gastric cancer

**DOI:** 10.1038/s41419-026-08627-y

**Published:** 2026-03-23

**Authors:** Xiaolin Xu, Han Wu, Jin Shang, Yating Wang, Yifan Yang, Tianying Cai, Lu Chen, Xuechun Xu, Chenyu Zhang, Wenqing Zhang, Daxuan Wang, Mingqing Zhang, Yan-yan Zhan

**Affiliations:** 1 Cancer Research Center, School of Medicine, Xiamen University, Xiamen, China; 2 Department of Geriatrics, Renji Hospital, Shanghai Jiao Tong University School of Medicine, Shanghai, China; 3 Department of Gastroenterology, School of Medicine, Xiamen University, Xiamen, China; 4 Department of Gastroenterology, The 909th Hospital, School of Medicine, Xiamen University, Zhangzhou, China; 5 College of Clinical Medicine, Fujian Provincial Hospital, Fuzhou, China

**Keywords:** Gastric cancer, Cell migration

## Abstract

Metastatic gastric cancer (GC) has a poor prognosis. Recent research demonstrated the aberrant expression of nuclear receptor HNF4α and the regulatory roles of its isoforms during the processes of tumorigenesis and development. However, the expression patterns of HNF4α and its potential as a therapeutic target in metastatic GC remain elusive. In this study, we unveiled that P2 promoter-driven HNF4α (P2-HNF4α) was highly expressed in distant metastasis of GC, playing a pivotal role in fostering the migration and metastasis of GC cells both in vitro and in vivo. The transactivational activity was essential for HNF4α to promote GC cell migration. An integrative analysis of transcriptome and metabolome implied the involvement of the glycolytic pathway in the promotion of GC cell migration by P2-HNF4α. We further found that P2-HNF4α directly bound to the enhancer of the HKDC1 gene and upregulated its expression, thereby orchestrating a metabolic rewiring conducive to promoting GC migration and metastasis. Mycophenolic acid, an active metabolite of the FDA-approved drug mycophenolate mofetil, demonstrated the ability to suppress HKDC1 expression and GC migration and metastasis in vitro and in vivo through antagonizing HNF4α. Therefore, this study sheds light on the HNF4α-HKDC1 axis as a key player in GC metastasis, providing a promising targeted therapeutic strategy for metastatic GC.

## Introduction

Gastric cancer (GC) ranks fifth globally, with approximately 1.1 million new cases and over 768,000 deaths (GLOBOCAN 2020) [1]. Early GC is often overlooked due to asymptomatic or atypical symptoms, resembling chronic conditions such as gastric ulcer and gastritis. Low early diagnostic rates, especially in East Asia, lead to advanced-stage discoveries. Advanced GC with metastasis poses a formidable challenge, characterized by a substantial incidence and mortality. Current therapeutic strategies, including surgery, chemotherapy, and a few targeted therapies, offer limited success in advanced stages. Addressing treatment resistance, refining early detection methods, and identifying new molecular targets for metastasis are crucial for improving outcomes in metastatic GC.

Hepatocyte Nuclear Factor 4 alpha (HNF4α) stands as a pivotal transcription factor within the nuclear receptor superfamily, which represents critical molecular targets for approximately 13% of FDA-approved drugs [2, 3]. Through the formation of homodimers and subsequent DNA binding, HNF4α triggers the transcription of downstream target genes involved in glucose, lipid, and bile acid metabolism, as well as hepatocyte differentiation [4–7]. The HNF4α gene encodes 12 isoforms driven by two promoters, with the P1 promoter generating isoforms α1–α6 and the P2 promoter producing isoforms α7–α12 through alternative splicing, which are proposed to orchestrate the transcription of distinct target genes to fulfill diverse biological roles [8–10]. Additionally, P1-HNF4α is expressed in the hepatocytes, intestine, kidney, and epididymis, whereas P2-HNF4α is predominantly distributed in the bile duct, intestine, pancreas, stomach, and epididymis [8].

Recent research has demonstrated the aberrant expression of HNF4α and its regulatory role during the processes of tumorigenesis and development across multiple cancers, including hepatocellular carcinoma, colorectal cancer, cervical cancer, and GC [11]. The expression of P1-HNF4α in hepatocytes underwent downregulation during the onset and progression of hepatocellular carcinoma, owing to the ability of P1-HNF4α to hinder epithelial-mesenchymal transition either by suppressing the transcription of downstream target genes like Snail, Slug, and HMGA or by impeding the β-catenin signaling pathway [12, 13]. In colorectal cancer, phosphorylation by the tyrosine kinase Src and subsequent degradation of the differentiation-promoting P1-HNF4α led to the attenuation of its anti-cancer properties [14]. Conversely, the upregulation of the proliferation-promoting P2-HNF4α encouraged the development of inflammation-associated colorectal cancer by promoting the expression of the pro-inflammatory protein RELMβ [9]. HNF4α mediated cervical cancer cell proliferation and tumor growth by activating the Wnt/β-Catenin signaling pathway [15]. Our recent study showed that P2-HNF4α drove lung invasive mucinous adenocarcinoma (IMA) growth and metastasis via a positive feedback loop involving P2-HNF4α, lncRNA BC200, and FMR1; mycophenolic acid (MPA), the active metabolite of FDA-approved drug mycophenolate mofetil (MMF), emerged as an HNF4α antagonist with anti-IMA properties [16]. In early GC, heightened expression of HNF4α triggers the activation of the Wnt/β-catenin signaling pathway by enhancing WNT5A expression, thereby facilitating GC growth [17]. The HNF4α-BAP31 axis promoted GC progression by binding to and stabilizing VDAC1, thereby synchronously regulating both cell proliferation and ferroptosis [18]. Therefore, HNF4α exerts context-dependent regulatory effects by engaging distinct target genes or mechanisms in different tumors or pathological processes. However, the expression and role of HNF4α in aggressively metastatic GC remain undefined.

In the present study, we uncovered that P2-HNF4α was highly expressed in distant metastasis of GC, significantly contributing to the migration and metastasis of GC cells both in vitro and in vivo. Further investigations revealed that P2-HNF4α upregulated the expression of HKDC1, thereby remodeling the metabolic program to regulate GC metastasis. Importantly, MMF demonstrated the ability to suppress HKDC1 expression by antagonizing HNF4α, thus mitigating GC migration and metastasis in both in vitro and in vivo models. This research offers promising insights into the treatment of metastatic GC and holds potential for improving patient survival rates.

## Results

### P2-HNF4α expression was up-regulated in human metastatic GC samples

**Fig. 1:**
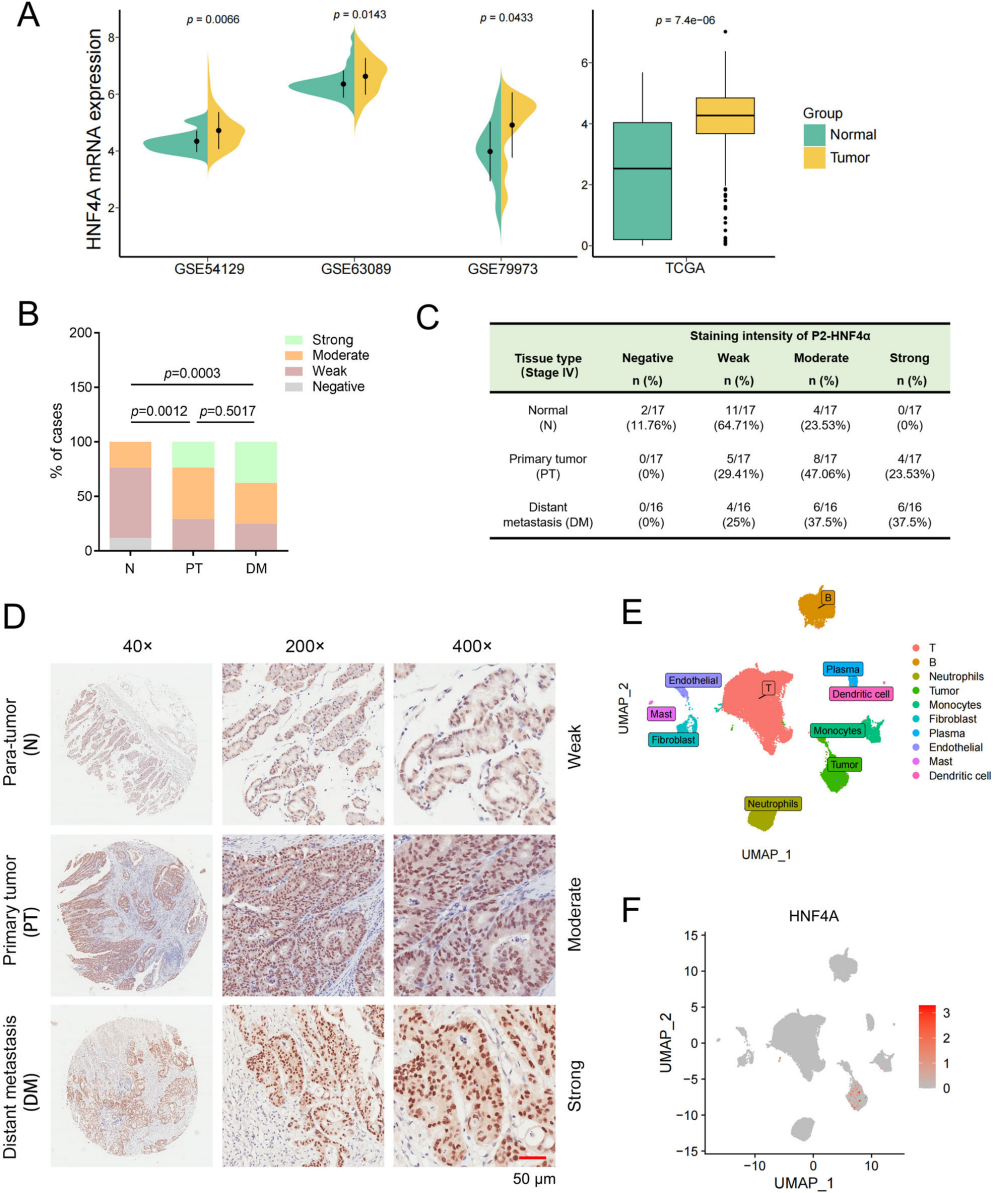
The expression of P2-HNF4α was elevated in human metastatic GC samples. **A** Assessment of HNF4α mRNA expression levels in gastric tissues and their corresponding normal counterparts from publicly available databases (left: GSE54129 (*n* = 21 for normal, *n* = 111 for tumor), GSE63089 (*n* = 45 for normal, *n* = 45 for tumor), and GSE79973 (*n* = 10 for normal, *n*= 10 for tumor) datasets in the GEO database; Right: TCGA database (*n* = 36 for normal, *n* = 408 for tumor)). **B**, **C** Statistical representation of P2-HNF4α expression levels (IHC staining intensity) in clinical specimens of metastatic GC patients, including paracancerous or normal gastric tissues (*N*, *n* = 17), primary tumors (PT, *n* = 17), and distant metastases (DM, *n* = 16). **D** Representative IHC staining images of P2-HNF4α in samples from the same GC patient, including para-tumor normal gastric tissue, primary tumor, and distant metastasis. Scale bar: 50 μm. **E** Uniform Manifold Approximation and Projection (UMAP) plot illustrating ten identified cell types from nine primary and metastatic GC samples (as indicated in Supplementary Fig. S1E). **F** Expression patterns of HNF4α in the ten identified cell types in single-cell data.

By analyzing the mRNA expression data from three GC-related datasets, GSE54129, GSE63089 and GSE79973, in the Gene Expression Omnibus (GEO) database and those in the Cancer Genome Atlas (TCGA) database, we confirmed the overexpression of HNF4α in GC tissues as compared to normal gastric tissues (Fig. 1A). To explore the possible role of HNF4α in GC, we analyzed the mRNA data from the GC cohort in the TCGA database, revealing the top 200 co-expressed genes of HNF4α, visually represented in a volcano plot (Supplementary Fig. S1A). Furthermore, Kyoto Encyclopedia of Genes and Genomes (KEGG) pathway analysis indicated that the genes associated with HNF4α might play a role in regulating carbohydrate metabolism and metastasis-related pathways, including tight junctions, motor proteins, and adherens junctions (Supplementary Fig. S1B).

To comprehensively elucidate the putative role of HNF4α in the metastatic progression of GC, we conducted an IHC analysis of P1- and P2-HNF4α expression in clinical specimens from metastatic GC patients. P2-HNF4α, rather than P1-HNF4α, was expressed in GC tissues and the paracancerous or normal gastric tissues; strikingly, the expression profile of P2-HNF4α exhibited an escalation in primary tumor lesions and a further augmentation in distant metastatic lesions of GC when juxtaposed with paracancerous or normal gastric tissues (Fig. 1B–D and Supplementary Fig. S1C and S1D). This observation implied a potential implication of P2-HNF4α in the metastatic cascade of GC. Noteworthy was the predominant expression of P2-HNF4α in tumor cells, rather than other types of cells, within the GC samples (Fig. 1D), a discernment substantiated through bioinformatics analysis utilizing single-cell sequencing data retrieved from human GC samples (GSE163558) within the GEO database (Fig. 1E, 1F and Supplementary Fig. S1E).

### P2-HNF4α promoted the migration and metastasis of GC cells in vitro and in vivo

**Fig. 2:**
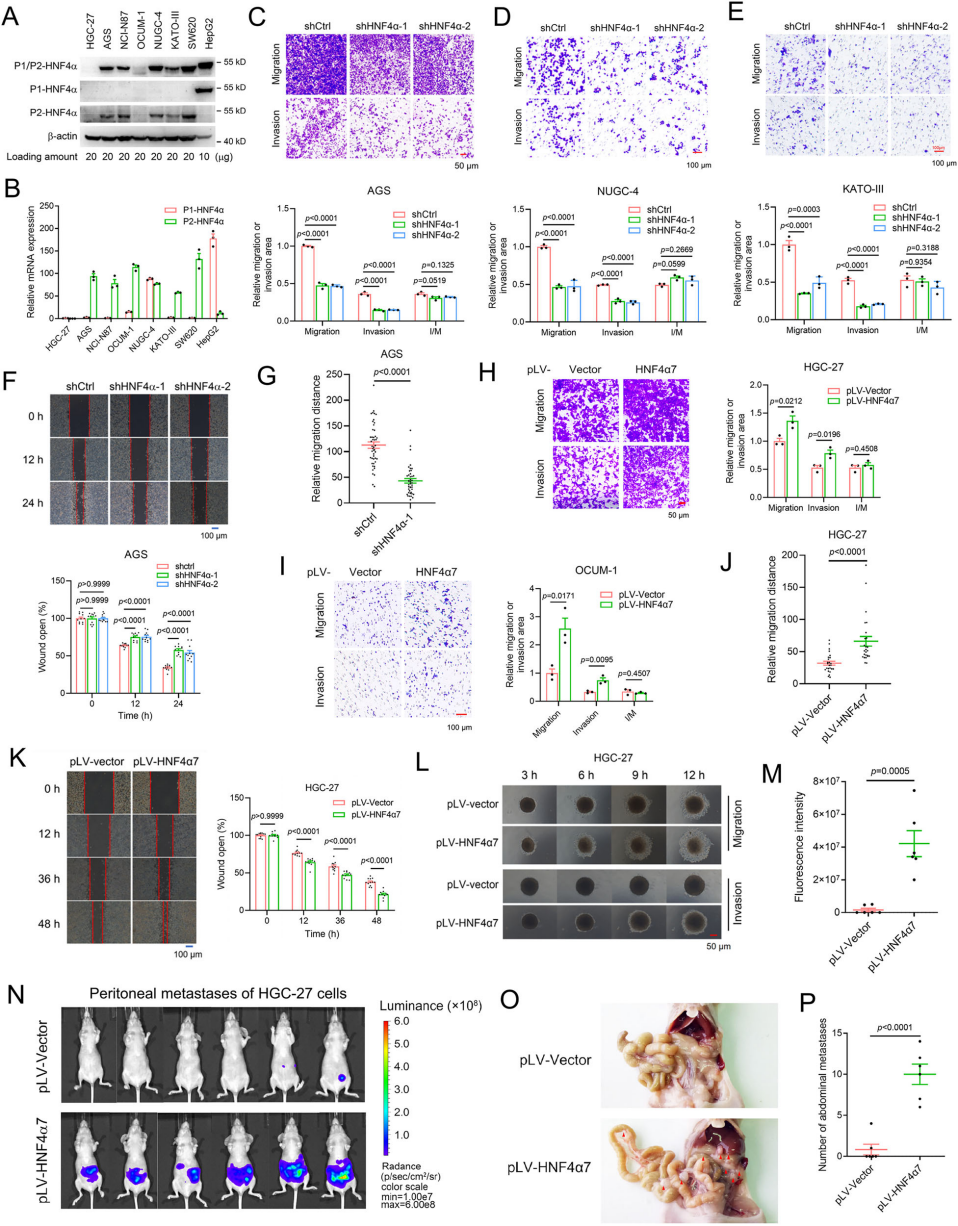
P2-HNF4α promoted the migration and invasion of GC cells in vitro. **A**, **B** P2-HNF4α but not P1-HNF4α was expressed in GC cell lines as assayed by WB (**A**) and real-time PCR (**B**). Immunodetection of P1/P2-HNF4α, P1-HNF4α and P2-HNF4α was accomplished using specific anti-HNF4α antibodies—namely, H1415, K9218 and H6939, respectively. **C**–**E** HNF4α knockdown decreased the migration and invasion abilities of AGS, NUGC-4, and KATO-III cells as examined by the transwell assay (scale bar: 50 μm or 100 μm as indicated). Representative images (upper panel) and relative migration or invasion area (lower panel) were shown (*n* = 3 biologically independent samples). I/M: ratio of invasion (I) to migration (M). **F** HNF4α knockdown inhibited the migration of AGS cells as detected by the wound healing assay (scale bar: 100 μm). Representative images (upper panel) and quantification of wound distance (lower panel) were shown (*n* = 11 biologically independent samples). **G** Single cell trajectory tracking demonstrated the reduced motility of AGS cells by HNF4α knockdown (*n* = 47 cells for shCtrl group, *n* = 42 cells for shHNF4α group. **H**, **I** Effects of HNF4α7 on the migration and invasion of HGC-27 and OCUM-1 cells in transwell assay (scale bar: 50 μm or 100 μm as indicated). Representative images (upper or left panel) and relative migration or invasion area (lower or right panel) were shown (*n* = 3 biologically independent samples). I/M: ratio of invasion (I) to migration (M). **J**–**L** Effects of HNF4α7 on the migration and invasion of HGC-27 cells in single cell trajectory tracking (**J**), wound healing assay (**K**) (scale bar: 100 μm), and 3D tumor spheroid migration and invasion assay (**L**) (scale bar: 50 μm). Migration distance (**J**), representative images (**K** (left panel), **L**), and quantification of wound distance (**K**, right panel) were shown (*n* = 25 cells for both the pLV-Vector group and pLV-HNF4α7 group in J; *n* = 12 biologically independent samples for K; n = 3 biologically independent samples for L). **M**, **P** Overexpression of HNF4α7 significantly promoted the peritoneal metastasis of GC cells. pLV-Vector and pLV-HNF4α7 sublines of HGC-27 cells were subjected to intraperitoneal injection into nude mice (6 mice per group). Thirty days later, quantification (**M**) and images (**N**) of luciferase expression, and representative images (**O**) and number (**P**) of metastatic nodules in the abdominal cavity of nude mice for each group were presented. All macroscopic metastatic lesions discernible by visual inspection were included in the quantitative analysis (**O**, **P**). Data were presented as the mean ± SEM. The difference in significance was analyzed by an unpaired two-tailed Student’s *t*-test (**B**, **D**). Data were presented as the mean ± SEM. The difference significance was analyzed by one-way ANOVA (**C**–**F**) or unpaired two-tailed Student’s *t*-test (**G**–**K**, **M**, **P**).

We then embarked on elucidating the role of P2-HNF4α in the metastatic processes of GC. GC exhibits high heterogeneity, being divided into four subtypes (chromosomal instability (CIN, ~50%), microsatellite instability (MSI, ~22%), genome stable (GS, ~20%), and Epstein–Barr virus-positive (EBV, ~8%)) based on their unique molecular characteristics [19]. Hence, the expression pattern of HNF4α was initially analyzed by western blot (WB) and real-time PCR in six human GC cell lines belonging to the three major molecular subtypes of GC, including CIN (HGC-27, NCI-N87, NUGC-4, and KATO-III cell lines), MSI (AGS cell line), and GS (OCUM-1 cell line) (Supplementary Fig. 2A). The distinctive expression patterns of P1-HNF4α and P2-HNF4α were observed in hepatocellular carcinoma HepG2 cells and colon cancer SW620 cells, aligning with previous reports [8] and thus validating the specificity of both the commercial antibodies and the designed DNA primers for HNF4α (Fig. 2A, B). Intriguingly, AGS, NCI-N87, OCUM-1, NUGC-4, and KATO-III cells exhibited exclusive expression of P2-HNF4α, while HGC-27 cells displayed no detectable expression of HNF4α (Fig. 2A, B). These results suggested that P2-HNF4α, rather than P1-HNF4α, was expressed across cell lines representative of the three predominant subtypes of GC.

Thereafter, AGS, NUGC-4, and KATO-III cells with high HNF4α expression and HGC-27 and OCUM-1 cells with negative or low HNF4α expression were chosen for subsequent HNF4α knockdown and overexpression experiments, respectively. Employing two distinct short hairpin RNAs targeting HNF4α (shHNF4α-1 and shHNF4α-2) for HNF4α knockdown in AGS, NUGC-4, and KATO-III cells resulted in a profound reduction in their migration and invasion capabilities, as assessed through transwell migration and invasion assays, wound healing assay, and single cell trajectory tracking assay (Fig. 2C–G and Supplementary Fig. S2B–D). Conversely, the overexpression of HNF4α7 (a widely recognized representative of the P2‑HNF4α isoforms) significantly augmented the migration and invasion capacities of HGC-27 and OCUM-1 cells in transwell, single cell trajectory tracking, wound healing, and three-dimensional (3D) tumor spheroid migration and invasion assays (Fig. 2H–L and Supplementary Fig. S2E, S2F). Throughout the duration of the aforementioned experiments (not exceeding 48 h), the viability or proliferation of these cell lines remained unaffected by HNF4α knockdown or overexpression (Supplementary Fig. S2G–K), confirming the regulation of P2-HNF4α on the migration and invasion of cell lines belonging to the three major subtypes of GC. Remarkably, the regulatory impact of P2-HNF4α on GC metastasis appeared to be predominantly manifested during the migration process, evidenced by comparable fold changes observed in transwell migration and invasion assays (Fig. 2C–E, H, I). Consequently, subsequent in vitro functional experiments were specifically directed towards cell migration assays.

Furthermore, we studied the effect of P2-HNF4α on GC metastasis in vivo. HNF4α7-overexpression and control HGC-27 cells (stably expressing luciferase, 1 × 10^7^ per mouse) were respectively injected into the abdominal cavity of nude mice, and the peritoneal metastasis was detected 30 days later using bioluminescence analysis. As shown in Fig. 2M, N, overexpression of HNF4α7 significantly promoted the peritoneal metastasis of human GC cells as compared with the control group. The number of metastatic nodules in the abdominal cavity was counted to confirm the promoting effect of P2-HNF4α on GC metastasis (Fig. 2O, P).

### Transactivational activity was required for HNF4α to promote GC cell migration

**Fig. 3:**
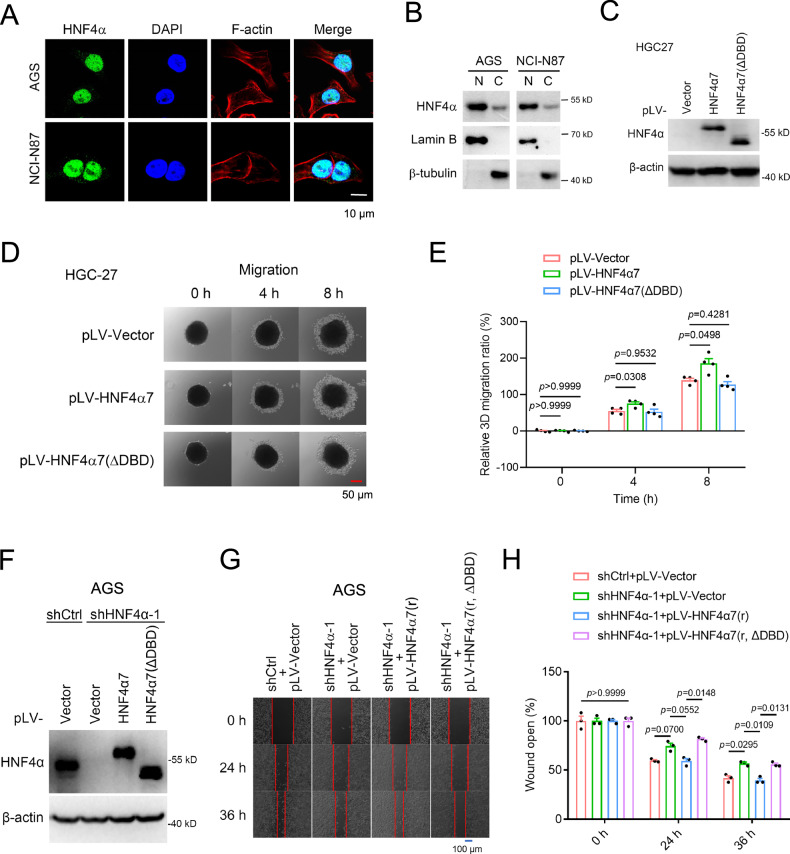
The transactivational activity of P2-HNF4α was required for the promotion of GC cell migration. **A**, **B** The subcellular localization of HNF4α in AGS and NCI-N87 cell lines was detected by IF assay (**A**) and nuclear/cytoplasmic fractionation assays with WB (**B**). The nucleus and cytoplasmic skeleton protein F-actin were labeled with DAPI (blue) and Rhodamine Phalloidinblue (red), respectively. Scale bar: 10 μm. Lamin B and β-tubulin served as reference markers for the nucleus and the cytoplasm, respectively. N nucleus; C cytoplasm. **C** Expression levels of transfected HNF4α7 and its mutant HNF4α7(ΔDBD) were assessed by WB analysis using anti-HNF4α (H1415) antibody. **D**, **E** Effects of HNF4α7 and HNF4α7(ΔDBD) on the migration of HGC-27 cells as determined by 3D tumor spheroid migration experiment. Representative images (**D**) and quantification (**E**) were shown (*n* = 4 biologically independent samples). Scale bar: 50 μm. **F**–**H** HNF4α7, rather than HNF4α7(ΔDBD), promoted the migration of AGS cells, as assessed by the wound healing assay. Representative images (**G**, scale bar: 100 μm) and quantification (**H**) were presented (*n* = 3 biologically independent samples). AGS cell sublines, shCtrl and shHNF4α, were transfected with pLV-vector, pLV-HNF4α7(r) or its mutant HNF4α7(r, ΔDBD). The HNF4α7 silence mutation HNF4α7(r), which could not be recognized by shHNF4α-1, was utilized in the RNAi rescue experiments (r indicated rescue). The expression level of HNF4α in each sample was examined by WB using anti-HNF4α (H1415) antibody (**F**). All data were presented as average ± SEM, and statistical significance was determined by one-way ANOVA.

To elucidate the mechanism by which P2-HNF4α facilitated the migration and metastasis of GC cells, our initial focus was on analyzing the subcellular localization of P2-HNF4α in both human clinical GC samples and immortalized GC cell lines. As illustrated in Fig. 1D, P2-HNF4α exhibited a predominant nuclear localization in clinical GC samples. The nuclear location of P2-HNF4α was corroborated in AGS and NCI-N87 cell lines through immunofluorescence (IF) and nuclear/cytoplasmic fractionation assays, as depicted in Fig. 3A, B.

Given that nuclear receptors play diverse roles through genomic effects (acting as transcription factors) or non-genomic signaling (physically interacting with various signaling proteins) [20], we delved into whether the transactivational activity of HNF4α was imperative for promoting the migration of GC cells. Overexpression of HNF4α7, but not its deletion mutant HNF4α7(ΔDBD), which lacked the ability to bind to the promoter of target genes, significantly enhanced the migration of HGC-27 cells (Fig. 3C–E). Moreover, the diminished migration of AGS cells observed upon HNF4α knockdown was restored by re-expressing HNF4α7, but not by the re-expression of the non-DNA-binding mutant HNF4α7(ΔDBD), as illustrated in Fig. 3F–H. This comprehensive set of experiments solidified our confirmation that P2-HNF4α relied on its transactivational activity to effectively promote the migration of GC cells.

### HNF4α orchestrated the upregulation of the glycolytic pathway in GC cells

**Fig. 4:**
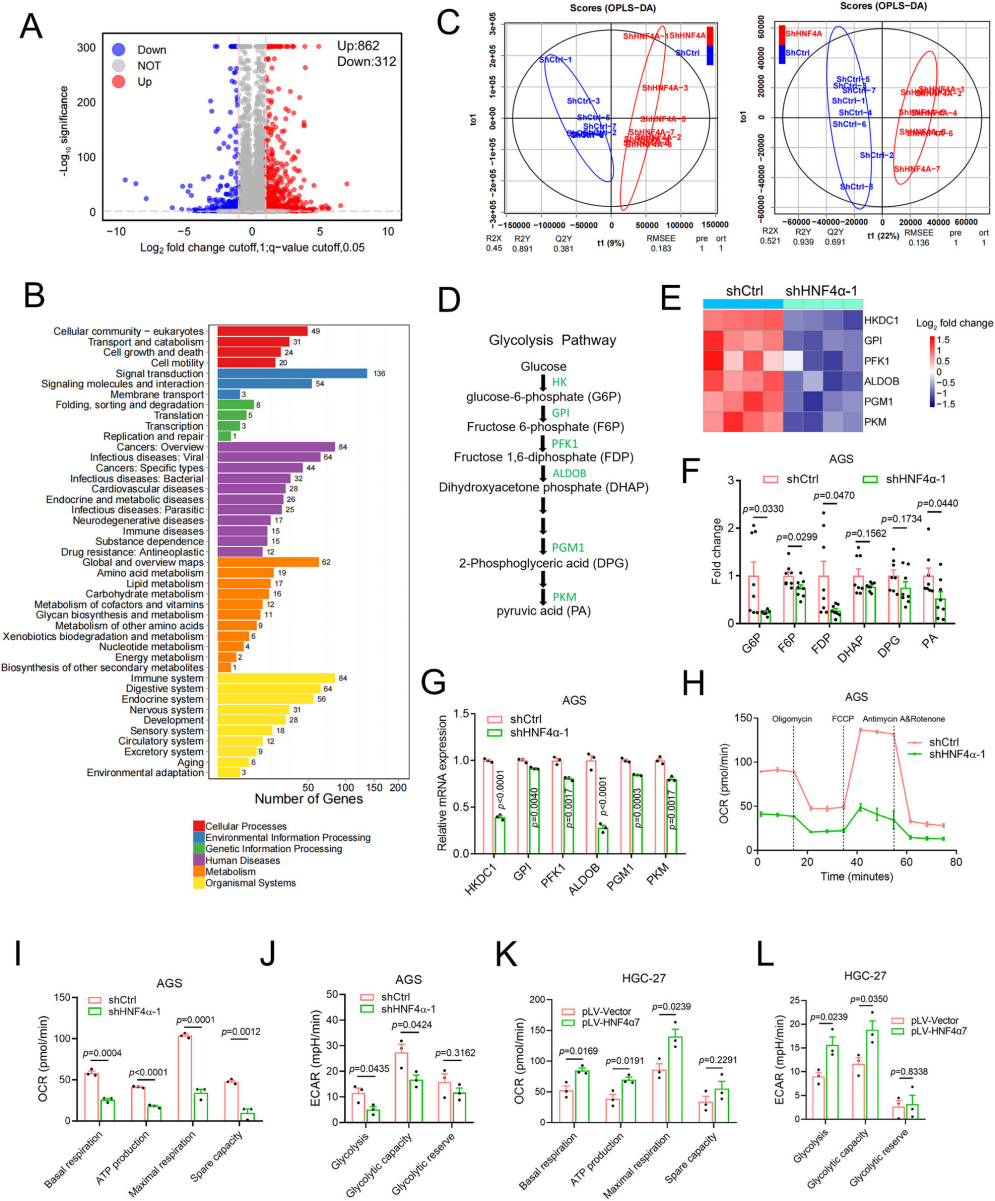
HNF4α orchestrated the upregulation of the glycolytic pathway in GC cells. **A** A volcano plot illustrated genes as up-regulated (Up), down-regulated (Down), or non-regulated (NOT) in AGS cells where endogenous HNF4α was knocked down, as revealed by transcriptional profiling (RNA-seq). **B** KEGG enrichment analysis of differentially expressed genes resulting from HNF4α knockdown in (**A**). **C** Metabolome analysis delineated the metabolic patterns modulated by HNF4α knockdown. On the left were results related to amino acid/lipid metabolism (cation mode), and on the right were those associated with sugar/nucleotide metabolism (anion mode). **D** Depiction of glycolysis metabolic pathways and the corresponding enzymes. HK hexokinase, GPI glucose-6-phosphate isomerase, PFK1 phosphofructokinase 1, ALDOB aldolase, PGM1 phosphoglycerate mutase 1, PKM pyruvate kinase isoenzyme 2. The green font indicated a decrease in AGS cells with endogenous HNF4α knocked down in RNA-seq from (**A**). **E** A heatmap showing glycolytic enzymes regulated in AGS cells with HNF4α knockdown in RNA-seq from (**A**). **F** A column chart demonstrated glycolytic metabolites regulated in AGS cells with HNF4α knockdown in metabolome analysis from (**C**) (*n* = 8 biologically independent samples). **G** Expression levels of glycolytic enzymes were down-regulated in AGS cells by HNF4α knockdown, as analyzed by real-time PCR. **H**–**J** Influence of HNF4α knockdown on OCR (**H**, **I**) and ECAR (**J**) of AGS cells. **K**, **L** Overexpression of HNF4α7 increased OCR (**K**) and ECAR (**L**) in HGC-27 cells. All data were presented as average ± SEM, and statistical significance was determined by unpaired two-tailed Student’s *t*-test (**F**, **G**, **I**–**L**).

To unravel the mechanisms underlying P2-HNF4α’s promotion of GC metastasis, we conducted transcriptional profiling (RNA-seq) on AGS cells with stably knocked-down HNF4α. Comparative analysis with control AGS cells (shctrl) revealed 862 upregulated and 312 downregulated genes in AGS shHNF4α cells (Fig. 4A). KEGG pathway analysis highlighted the involvement of these differentially expressed genes in the regulation of cellular processes, genetic information processing, metabolism, and other pathways (Fig. 4B).

Given the pivotal role of metabolic remodeling during tumor cell migration [21, 22], we delved into an untargeted metabolome analysis to pinpoint the specific metabolic pathways affected by HNF4α knockdown. As illustrated in Fig. 4C, amino acid/lipid metabolism (cation mode, left panel) and sugar/nucleotide metabolism (anion mode, right panel) underwent significant changes following HNF4α knockdown in AGS cells. An integrated analysis of the transcriptome and metabolome uncovered diminished levels of glycolytic enzymes (HKDC1, GPI, PFK1, ALDOB, PGM1, and PKM) and their corresponding metabolites (glucose-6-phosphate (G6P), fructose-6-phosphate (F6P), FDP, dihydroxyacetone phosphate (DHAP), 2,3-bisphosphoglyceric acid (DPG), and pyruvate (PA)) upon HNF4α knockdown in the glycolytic pathway (Fig. 4D–F). Real-time PCR verification confirmed HNF4α’s regulatory influence on the mRNA levels of these glycolytic enzymes (Fig. 4G).

Further investigations revealed that HNF4α knockdown decreased the oxygen consumption rate (OCR), ATP production, and extracellular acidification rate (ECAR) in AGS cells (Fig. 4H–J). Conversely, overexpression of HNF4α7 increased these indices in HGC-27 cells (Fig. 4K, L), affirming the positive regulatory impact of P2-HNF4α on glycolysis and the following lactate generation and oxidative phosphorylation.

### P2-HNF4α induced HKDC1 expression to promote the migration of GC cells

**Fig. 5:**
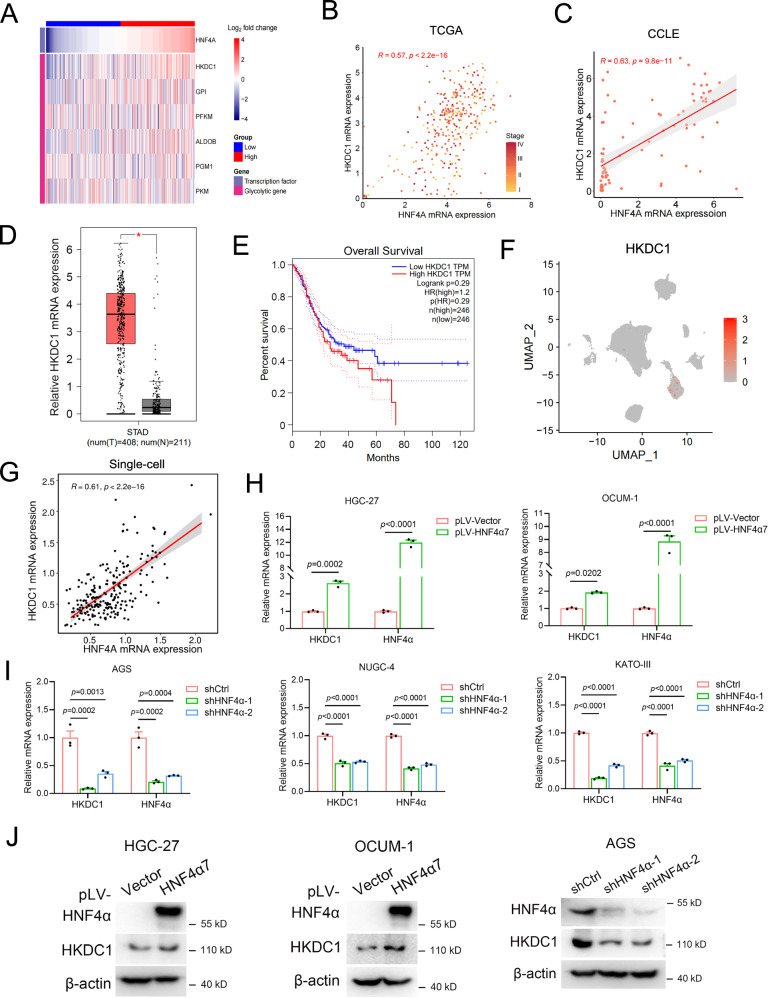
P2-HNF4α induced HKDC1 expression to promote the migration of GC cells. **A** A heatmap depicted the mRNA expression levels of glycolytic genes (as mentioned in Fig. 5E, **G** in high-expression and low-expression HNF4α groups within the TCGA GC cohort. The median value of HNF4α was utilized as the cutoff threshold to categorize GC patients into high-expression and low-expression HNF4α groups. **B** A scatter plot illustrated the correlation between HNF4α and HKDC1 mRNA expression in TCGA GC samples (*n* = 408, Spearman’s correlation test). **C** Another scatter plot displayed the correlation between HNF4α and HKDC1 mRNA expression in CCLE GC cell lines (*n* = 71, Spearman’s correlation test). **D** A boxplot presented HKDC1 mRNA expression in GC tissues and the corresponding normal gastric tissues in TCGA and Genotype-Tissue Expression (GTEx) databases (*n* = 408 for tumor and *n* = 211 for normal) using GEPIA (http://gepia.cancer-pku.cn/). **E** Assessment of the prognostic values of overall survival in GC patients based on the mRNA expression level of HKDC1 via Kaplan–Meier plotter by GEPIA (cutoff: high HKDC1 60%, low HKDC1 40%). **F** The expression profiles of HKDC1 across the ten identified cell types derived from the single-cell data referenced in Fig. 1E and Supplementary Fig. S1E were depicted. **G** A scatter plot illustrated the correlation between HNF4α and HKDC1 mRNA expression in single-cell RNAseq datasets, as determined by Spearman’s correlation test. **H**, **I** The expression level of HKDC1 was upregulated in HGC-27 and OCUM-1 cells by HNF4α7 overexpression and down-regulated in AGS, NUGC-4, and KATO-III cells by HNF4α knockdown, as analyzed by real-time PCR. **J** The expression level of HKDC1 was upregulated in HGC-27 and OCUM-1 cells by HNF4α7 overexpression and down-regulated in AGS cells by HNF4α knockdown, as analyzed by WB. Data were presented as the mean ± SEM. The difference in significance was analyzed by unpaired two-tailed Student’s *t*-test (**H**) or one-way ANOVA (**I**).

To pinpoint the pivotal regulator mediating HNF4α-driven metabolic remodeling and migration in GC cells, we conducted an analysis to identify which of the aforementioned glycolytic enzymes exhibited a positive correlation with HNF4α expression in TCGA GC samples. Among the six glycolytic enzymes, HKDC1 displayed the most significant difference in expression between HNF4α-high and HNF4α-low GC samples (Fig. 5A). The positive correlation between HKDC1 and HNF4α expression was confirmed in TCGA GC samples (Fig. 5B, *n* = 408, rho = 0.57, *p* = 2.2 × 10^−16^) and in cancer cell line encyclopedia (CCLE) GC cell lines (Fig. 5C, *n* = 71, rho = 0.63, *p* = 9.8 × 10^−11^).

Further analysis of mRNA expression data in the TCGA and GTEx databases revealed an elevation in HKDC1 mRNA expression in GC tissues compared to normal gastric tissues (Fig. 5D). Kaplan–Meier survival analysis demonstrated that the high HKDC1 expression group had a shorter overall survival than the low HKDC1 expression group in the TCGA database (Fig. 5E). Similar to HNF4α, HKDC1 demonstrated predominant expression within tumor cells in GC samples, displaying a positive correlation with HNF4α at the single-cell level, as evidenced by bioinformatics analysis of single-cell sequencing data from human GC samples (GSE163558) (Figs. 1E, F, 5F, G and Supplementary Fig. S1E). The regulatory influence of HNF4α on HKDC1 expression was validated by real-time PCR and WB. Stable overexpression of HNF4α7 in HGC-27 and OCUM-1 cells enhanced HKDC1 expression, while HNF4α knockdown using two shRNAs against HNF4α in AGS, NUGC-4, and KATO-III cells significantly decreased HKDC1 expression (Fig. 5H–J).

**Fig. 6:**
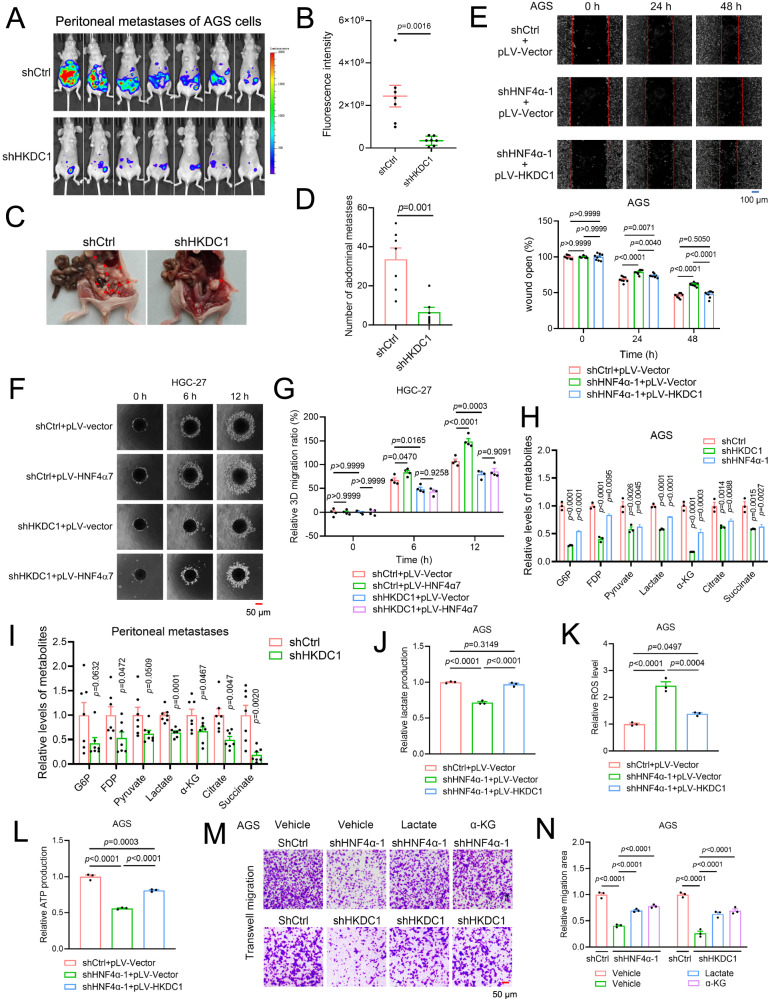
HKDC1 was the downstream target for HNF4α to promote GC metastasis. **A**–**D** HKDC1 knockdown significantly suppressed the peritoneal metastasis of GC cells. shCtrl and shHKDC1 sublines of AGS cells were subjected to intraperitoneal injection into nude mice (seven mice per group). Forty days later, images (**A**) and quantification (**B**) of luciferase expression, and representative images (**C**) and number (**D**) of metastatic nodules in the abdominal cavity of nude mice for each group were presented. **E** The diminished migratory capacity of AGS cells resulting from HNF4α knockdown was restored by HKDC1 overexpression, as demonstrated by the wound healing assay (scale bar: 100 μm). Representative images (upper panel) and quantification of wound distance (lower panel) were presented (*n* = 9 biologically independent samples). **F**, **G** HKDC1 knockdown attenuated the effects of HNF4α7 overexpression on the migration of HGC-27 cells by the 3D tumor spheroid migration assay. Representative images (**F**, scale bar: 50 μm) and quantification of wound distance (**G**) were shown (*n* = 4 biologically independent samples). **H** Metabolite levels of organic acid compounds (including glycolysis/TCA cycle Intermediates) before and after HNF4α or HKDC1 knockdown in AGS cells were assayed by targeted mass spectrometry (*n* = 3 biologically independent samples). **I** Metabolite levels of organic acid compounds (including glycolysis/TCA cycle Intermediates) of peritoneal metastases in each mouse of Fig. 6A were assayed by targeted mass spectrometry (seven mice per group). **J**–**L** The effects of HNF4α knockdown on lactate production (**J**), ROS levels (**K**), and ATP production (**L**) were restored by HKDC1 overexpression in AGS cells (*n* = 3 biologically independent samples). **M**, **N** The diminished transwell migration ability of AGS cells caused by HNF4α or HKDC1 knockdown was rescued by treatment with lactate (5 mM) or α-KG (5 mM). Representative images (**M**) and quantification (**N**) were shown (*n* = 3 biologically independent samples). Scale bar: 50 μm. Data were presented as the mean ± SEM. The difference significance was analyzed by unpaired two-tailed Student’s *t*-test (**B**, **D**, **I**) or one-way ANOVA (**E**, **G**, **H**, **J**–**L**, **N**).

Subsequently, we investigated the effect of HKDC1 on the migration potential of GC cells in vitro *and* in vivo. As indicated by the wound healing assay, knockdown of HKDC1 suppressed the migration of AGS cells, while overexpression of HKDC1 enhanced the migration of HGC-27 cells (Supplementary Fig. S3A–D). Notably, within the observed period of not more than 48 h, the growth or survival of these cell lines was not influenced by HKDC1 knockdown or overexpression (Supplementary Fig. S3E, S3F). In vivo studies confirmed that HKDC1 knockdown significantly suppressed peritoneal metastasis of AGS cells in mice, as demonstrated by bioluminescence imaging and increased metastatic nodule counts (Fig. 6A–D). Therefore, these results authenticated the positive regulation of HKDC1 on GC cell migration.

To assess whether the promotive effect of HNF4α on GC cell migration depends on HKDC1, we employed two complementary strategies—either overexpressing HKDC1 in HNF4α-knockdown AGS cells or overexpressing HNF4α in control and HKDC1-knockdown HGC-27 cells. A rescue phenotype on cell migration ability was observed when HKDC1 was overexpressed in AGS cells with HNF4α knockdown in a wound healing assay (Fig. 6E and Supplementary Fig. S3G). Moreover, the abilities of HNF4α7 to promote the migration of the HGC-27 cell line were impaired when HKDC1 was knocked down, as indicated by 3D tumor spheroid migration assays (Fig. 6F, G and Supplementary Fig. S3H). These findings strongly indicated that P2-HNF4α promoted GC cell migration by inducing HKDC1 expression.

We then investigated whether P2-HNF4α regulated glycolysis and the subsequent lactate generation and oxidative phosphorylation to promote GC cell migration via HKDC1 mediation. Results from targeted mass spectrometry assays of glucose metabolites indicated that the knockdown of HKDC1 or HNF4α resulted in a decrease in metabolites related to glycolysis and oxidative phosphorylation, including G6P, fructose-1,6-diphosphate (FDP), PA, lactate, α-ketoglutarate (α-KG), citrate, and succinate in AGS cells in vitro and/or in vivo (Fig. 6H, I). Importantly, effects of HNF4α knockdown on lactate production, reactive oxygen species (ROS) level (an indicator for oxidative phosphorylation), and ATP synthesis were effectively rescued by the forced expression of HKDC1 (Fig. 6J–L and Supplementary Fig. S3I). Furthermore, the migratory capacity of GC cells, which was impaired by the knockdown of either HNF4α or HKDC1, could be restored by the supplementation of lactate or α-KG in the culture media, as evidenced by transwell migration assays (Fig. 6M, N). Collectively, these findings underscore the pivotal role of the HNF4α-HKDC1 axis in sustaining the metabolic and energetic status of GC cells, thereby promoting their migration.

### HKDC1 was a transcriptional target of HNF4α

**Fig. 7:**
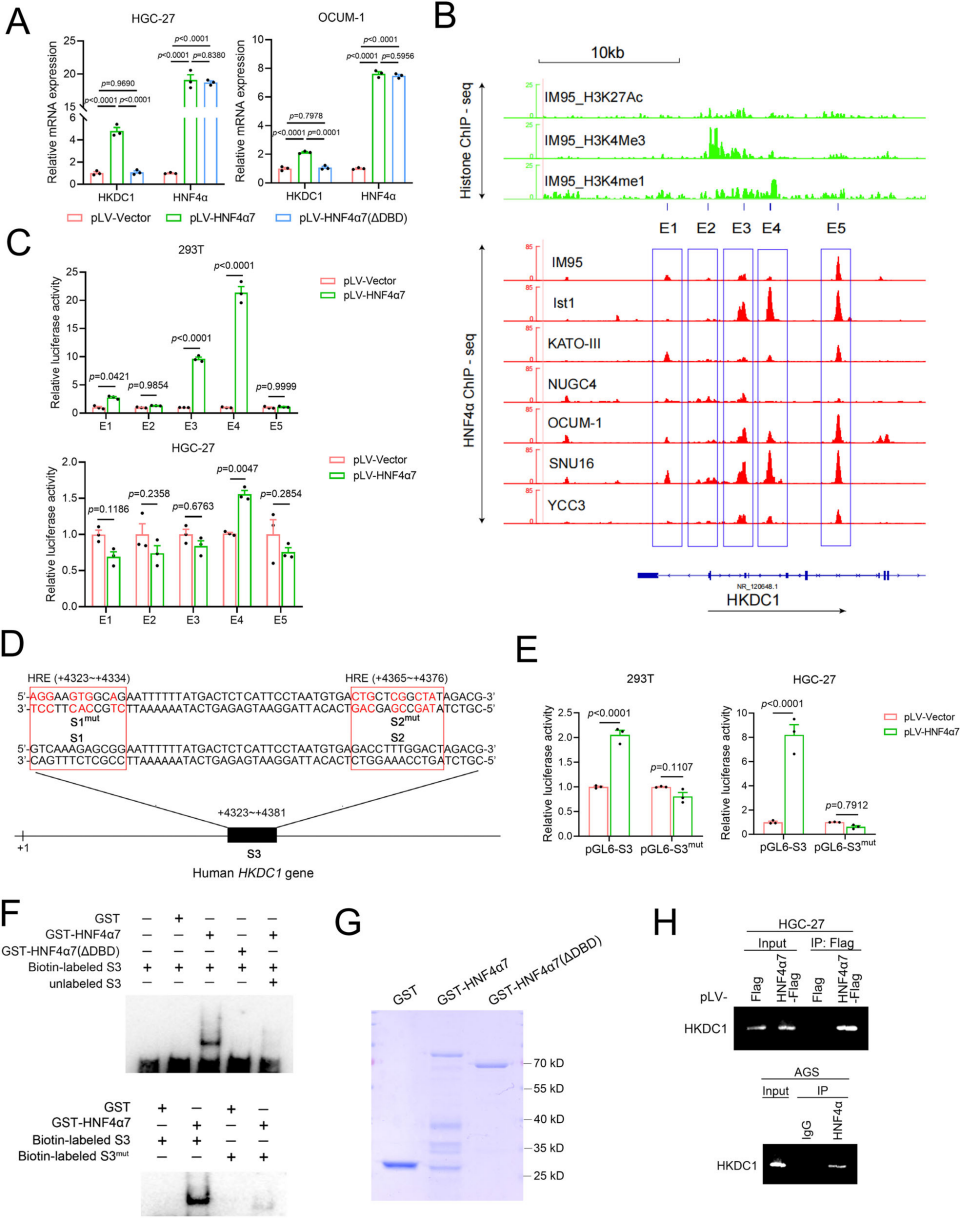
HKDC1 was a downstream transcription target of HNF4α. **A** Overexpression of HNF4α7, but not HNF4α7(ΔDBD), resulted in an elevation of HKDC1 mRNA expression in HGC-27 and OCUM-1 cells. **B** Integration of HNF4α ChIP-seq data in seven HNF4α-positive GC cell lines (IM95, Ist1, KATO-III, NUGC4, OCUM-1, SNU16, and YCC3) and histone ChIP-seq data (H3K27ac, H3K4me3, and H3K4me1) in IM95 cell line from the GEO database (GSE114018 and GSE75898) was performed at human *HKDC1* gene locus (NR_120648.1). The arrow indicated the direction of transcription. The blue frames denoted potential HNF4α binding regions, namely E1, E2, E3, E4 and E5. **C** HNF4α promoted the transcription of the potential HNF4α binding region E4. HEK293T cells transiently expressing or HGC-27 cells stably expressing pLV vector or pLV-HNF4α7 were cotransfected with pRL-TK and pGL6-TA constructs containing E1, E2, E3, E4, or E5, as indicated. Twenty-four hours later, cells were harvested, and a dual-luciferase reporter assay was conducted. **D** Two potential HNF4α response elements (HREs), namely S1 ( + 4323 ~ +4334 bp) and S2 ( + 4365 ~ +4376 bp), were predicted in the potential HNF4α binding region E4 within the human *HKDC1* gene locus. The sequence (+4323 ~ +4381 bp, named S3), containing S1 and S2, was employed in subsequent experiments. **E** Overexpression of HNF4α7 promoted the transcription of S3, but not its mutant with mutations in both S1 and S2 mentioned in (**D**). HEK293T cells transiently expressing or HGC-27 cells stably expressing pLV vector or pLV-HNF4α7 were cotransfected with pRL-TK and pGL6-TA constructs containing S3 or its mutant, as indicated. **F**–**H** The binding of HNF4α to S3 within the human *HKDC1* gene locus was detected by EMSA (**F**) and ChIP (**H**) assay. For EMSA, recombinant GST-HNF4α7 or its mutant GST-HNF4α7(ΔDBD) protein was incubated with biotin-labeled oligonucleotides of S3. Coomassie blue staining of the purified recombinant GST-HNF4α7 and GST-HNF4α7(ΔDBD) proteins is indicated in (**G**). For ChIP, HGC-27 cells (with HNF4α7 overexpressed beforehand) or AGS cells were harvested, and the ChIP assay was carried out. All data were shown as the mean ± SEM. The difference significance was analyzed by one-way ANOVA (**A**) or unpaired two-tailed Student’s *t*-test (**C**, **E**).

**Fig. 8:**
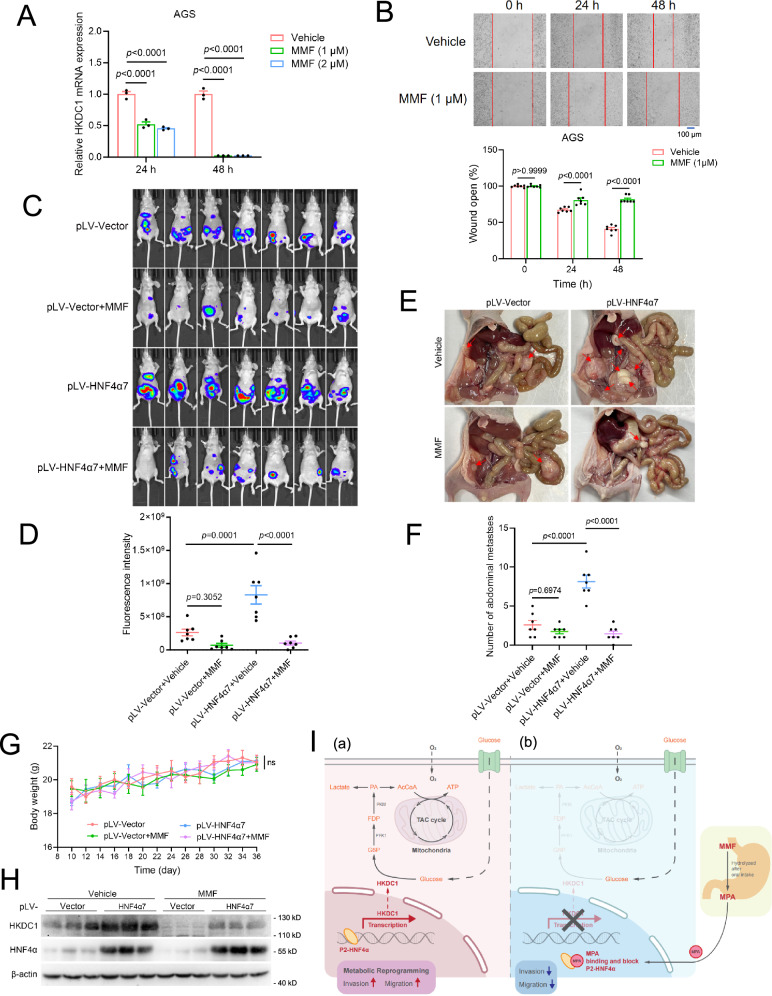
MMF inhibited the migration and metastasis of GC cells by antagonizing HNF4α. **A**, **B** The suppressive effects of MMF on the mRNA expression level of HKDC1 and the migration of AGS cells by real-time PCR (**A**) and wound healing assay (**B**). Representative images (**B**, upper panel) and quantification of wound distance (**B**, lower panel) were presented (*n* = 7 biologically independent samples). Scale bar: 100 μm. **C**–**H** MMF significantly suppressed the peritoneal metastasis of HNF4α-overexpressed HGC-27 cells, but not that of HGC-27 cells with minimal HNF4α expression in nude mice. One of the HGC-27 pLV-Vector and pLV-HNF4α7 cell sublines (all stably expressing luciferase, 1 × 10^7^ cells per mouse) was intraperitoneally injected into the nude mice. Ten days later, the mice were randomly divided into two groups (*n* = 7 per group), and treated with vehicle (0.9% NaCl) or MMF (40 mg/kg) once daily for 26 days by oral administration. Presented were images (**C**) and quantification (**D**) of luciferase expression, representative images (**E**) and number (**F**) of metastatic nodules in the abdominal cavity, and body weight curve (**G**) of nude mice for each group. All macroscopic metastatic lesions discernible by visual inspection were included in the quantitative analysis (**E**, **F**). The expression levels of HKDC1 and HNF4α in each group were examined by WB (**H**). **I** A proposed a working model for HNF4α-HKDC1 in the metabolism and metastasis of GC, which was antagonized by MPA. Data were presented as the mean ± SEM. The significance of differences was analyzed by one-way ANOVA (**A**, **D**, **F**, **G**) or unpaired two-tailed Student’s *t*-test (**B**).

We then delved into understanding how HNF4α increased HKDC1 expression. The overexpression of HNF4α7, but not HNF4α7(ΔDBD), led to an increase in the mRNA expression of HKDC1 in HGC-27 and OCUM-1 cells (Fig. 7A). To establish HKDC1 as a direct transcriptional target of HNF4α, we analyzed HNF4α ChIP-seq and histone ChIP-seq (H3K27ac, H3K4me3, and H3K4me1) data using seven HNF4α-positive GC cell lines (IM95, Ist1, KATO-III, NUGC4, OCUM-1, SNU16, and YCC3) from the GEO database (GSE114018 and GSE75898). This analysis revealed five ubiquitous HNF4α binding sites (namely Elements E1–E5) at the HKDC1 promoter, as indicated by positive H3K27ac, H3K4me3, and H3K4me1 signals (Fig. 7B). Each of the binding sites (E1–E5) was cloned into the luciferase reporter vector pGL6-TA, and the luciferase reporter assay results demonstrated that the transcriptional activity of E4 was specifically increased by the overexpression of HNF4α7 in 293 T and HGC-27 cells (Fig. 7C). Through sequence analysis, two conserved HNF4α response elements (HREs) were predicted at +4323 ~ +4334 bp (named S1) and +4365 ~ +4376 bp (named S2) within E4 (Fig. 8D). The sequence (+4323 ~ +4381 bp, named S3) containing S1 and S2, or its mutant (S3^mut^) with mutations in both S1 and S2 (S1^mut^ and S2^mut^) (Fig. 7D) failing to bind HNF4α, were then constructed into pGL6-TA. The transcriptional activity of S3, but not its mutant, was elevated by the overexpression of HNF4α7 in HGC-27 cells (Fig. 7E). Direct binding of HNF4α to S3 at the HKDC1 promoter was confirmed by electromobility shift assay (EMSA). The results showed that biotin-labeled wild-type S3 probes, but not the mutated S3 probes, formed complexes with recombinant HNF4α7 protein; the specificity of their binding was confirmed by the addition of excess unlabeled S3 probes (Fig. 7F, G). The interaction between HNF4α and the HKDC1 promoter in vivo was further validated in HGC-27 cells (with HNF4α7 overexpressed beforehand) and AGS cells through a ChIP assay (Fig. 7H). These findings conclusively identified HKDC1 as a novel transcriptional target of HNF4α.

### MPA antagonized HNF4α to suppress GC metastasis

Based on our previous study that identified MPA, the active metabolite of the FDA-approved drug MMF, as an HNF4α antagonist [16], we sought to assess whether the function of HNF4α could be antagonized by MMF in GC cells. Results from real-time PCR, wound healing, and transwell migration assays demonstrated that MMF suppressed the mRNA expression of HKDC1 and inhibited cell migration in HNF4α-high AGS, NUGC-4, and KATO-III cells rather than in HNF4α-low OCUM-1 cells (Fig. 8A, B and Supplementary Fig. S4A, S4B).

The inhibitory effects of MMF on HKDC1 expression and GC cell metastasis were further validated in vivo. Not surprisingly, the administration of MMF did not exert a significant inhibitory effect on the peritoneal metastasis of HGC-27 cells with extremely low HNF4α expression in nude mice. Strikingly, when HNF4α7 was overexpressed, not only did it greatly enhance the peritoneal metastasis of HGC-27 cells in nude mice, but it also endowed MMF with the potential to inhibit peritoneal metastasis in these cells (Fig. 8C, D). Consistently, the expression of HKDC1 in these peritoneal tumors was greatly suppressed by MMF only in the group with HNF4α7 overexpression (Fig. 8E). During the observation period, the administration of MMF did not exert a significant influence on the body weight of nude mice (Fig. 8F). Collectively, these findings indicated that MPA, the active metabolite of MMF, antagonized HNF4α to inhibit HKDC1 expression and metastasis of GC.

## Discussion

HNF4α is a context-dependent transcriptional regulator implicated in various cancers [9, 11, 15, 16, 18]. While it governs a broad spectrum of target genes—including the HNF4A–BAP31–VDAC1 axis in GC proliferation [18] and the HNF4α–BC200–FMR1 axis in invasive mucinous lung adenocarcinoma [16]—its role in metastatic GC remained unclear, hindering therapeutic development. In this study, we demonstrated that P2-HNF4α is upregulated in distant metastases and promotes GC migration and metastasis in vitro and in vivo (Figs. 1 and 2). Notably, its effect appeared more pronounced in migration than in matrix degradation, as suggested by comparable fold changes in Transwell migration and invasion assays (Fig. 2). The transactivational activity of HNF4α was essential for this migration phenotype, mirroring its role in early-stage GC proliferation (Fig. 3). Integrated transcriptomic and metabolomic profiling, combined with functional studies, revealed that P2-HNF4α transcriptionally activates HKDC1, leading to metabolic reprogramming that facilitates metastasis (Figs. 4–7). Although a transcription factor typically regulates multiple genes, its specific functional outputs—such as metastasis—are often primarily mediated by a single downstream target or a limited subset of critical targets. Thus, despite the multiplicity of HNF4α targets such as BAP31 and BC200 [16, 18], our data decisively positioned HKDC1 as the principal mediator of HNF4α-driven metastasis in GC. Importantly, the FDA-approved drug MMF—previously identified by us as an HNF4α antagonist [16]—effectively suppressed HKDC1 expression and GC metastasis in vitro and in vivo by targeting HNF4α (Fig. 8), suggesting a clinically translatable strategy. These findings were consistent across six cell lines representing the three major GC subtypes (~92% of cases), supporting the broad relevance of the HNF4α–HKDC1 axis and MMF as a potential anti-metastatic agent for most GCs. A limitation, however, is that the role of this axis in EBV-positive GC (~8% of cases) and its susceptibility to MMF remain unclear and merit future investigation.

Cancer metastasis stands as the primary cause of cancer-associated mortality. Hence, delineating the regulatory mechanisms governing the directed migratory phenotype of tumor cells is paramount. The dysregulation of cell migration during cancer progression dictates the propensity of tumor cells to evade primary sites and infiltrate neighboring tissues, ultimately seeding metastases. The metabolic reprogramming of cancer cells, known as the Warburg effect—transitioning from predominantly oxidative metabolism to glycolysis and generating lactate even in oxygen-replete environments, supports the high demands for energy, biosynthesis, and acidic microenvironment needed to maintain the malignant cancer cell state, including proliferative and metastatic phenotypes [21, 23]. Nevertheless, the heightened aerobic glycolysis observed in cancer cells is frequently misconstrued as a substitution for mitochondrial respiration, leading to an erroneous interpretation of respiratory impairment rather than dysregulation of glycolysis; in reality, numerous malignancies manifest the Warburg effect concurrently with the preservation of mitochondrial respiration [24–26]. HNF4α has been reported to regulate ‘metabolic switch’ characteristic of a proliferative phenotype in GC, through its target WNT5A to induce ‘Warburg effect’ anaerobic metabolism or IDH1 to enhance tricarboxylic acid (TCA) cycle metabolism, redox balance, and bioenergetics [17, 27], suggesting the importance of both the Warburg effect and mitochondrial respiration for GC growth. Here, we identified HKDC1 as a direct HNF4α target gene, essential for GC cell migration and metastasis both in vitro and in vivo. Given that HKDC1 governs the initial step of glucose metabolism, its expression level may impact glycolysis, lactate production, and oxidative phosphorylation. This phenomenon is indeed evident in our current investigation, wherein the knockdown of HKDC1 or its upstream regulator, HNF4α, effectively reduced metabolites related to glycolysis and oxidative phosphorylation and thereby attenuated GC cell migration and proliferation (Figs. 4–6 and Supplementary Figs. S2 and S3). Moreover, beyond its role in providing metabolic energy and fostering an acidic environment conducive to metastasis, lactate has been identified in recent research as a substrate for protein lactylation, playing a pivotal regulatory role in tumor metastasis [28]. Consequently, our study highlighted the HNF4α-HKDC1 axis as a significant target for GC metastasis, with the potential that pharmacological inhibitors targeting HNF4α or HKDC1 could suppress the metastasis of GC. Notably, positive correlations between HNF4α and HKDC1 expression were also observed in normal gastric samples (Supplementary Fig. S5A), albeit at much lower levels compared to those observed in GC samples (Supplementary Fig. S5B). This observation implies that the regulation of glycolysis and the TCA cycle by HNF4α may potentially be co-opted and magnified to advance GC progress.

HKDC1 emerges as a significant player in the orchestration of glucose homeostasis and lipid metabolism, acting as a fifth hexokinase [29–32]. Heightened levels of HKDC1 correlate with augmented glycolytic activity and enhanced cell proliferation in colorectal cancer cells, while elevated HKDC1 expression escalates glycolysis and boosts tumorigenesis in lung adenocarcinoma [33, 34]. HKDC1 ablation halts the development and progression of liver cancer by its interaction with the mitochondria, promoting metabolic reprogramming and redirecting glucose flux away from the TCA cycle [35]. An intriguing revelation from a recent study suggests that HKDC1 reshapes lipid metabolism to augment GC metastasis and foster resistance to cisplatin through the formation of a ribonucleoprotein complex [36]. In fact, their untargeted metabolomic profiling unveiled a significant impact of HKDC1 overexpression on both lipid metabolism and glucose metabolism in GC [36]. Together with our present study, these findings underscore the pivotal role of HKDC1 in regulating both glucose and lipid metabolism to promote GC progression and drug resistance.

Our findings reveal the critical role of the HNF4α–HKDC1 signaling axis in GC metastasis, identifying both molecules as potential therapeutic targets. However, a comprehensive review of the literature indicates that no well-validated, specific inhibitors of HKDC1 are currently available. Although one study listed twelve compounds as putative HKDC1 inhibitors [37]—including NSC 622190 (targeting RRM2), NSC 658387 (targeting RAD51), and NSC 651084 (targeting KIF11), among others—these compounds are in fact known to act primarily on other established targets and lack specificity for HKDC1. Therefore, the development of selective HKDC1 inhibitors remains a substantial challenge, requiring extensive preclinical optimization with uncertain outcomes. In contrast, we have previously identified an HNF4α antagonist, MPA derived from an FDA-approved drug MMF [16], presenting a viable repurposing opportunity with a clearer path toward clinical translation. Both in vitro and in vivo experiments confirmed that MMF suppressed GC metastasis by targeting HNF4α (Fig. 8), underscoring its potential therapeutic utility. While targeting upstream regulators such as HNF4α may entail broader biological effects than targeting downstream effectors like HKDC1, the current absence of specific HKDC1 inhibitors renders HNF4α a more immediately actionable target. Furthermore, as a repurposed agent, MMF has a well-established clinical safety profile and has also been reported to inhibit GC growth [38], thereby offering a dual therapeutic advantage.

In fact, numerous in vitro and in vivo preclinical experiments consistently manifest the anticancer properties of MPA against diverse malignancies [39–43]. However, two miniature clinical trials (*n* = 11 or 12 patients) investigating its anticancer efficacy in humans have yielded unsatisfactory results [44, 45]. Besides, not all cancer cell lines exhibit susceptibility to the anti-tumor effect of MPA [46]. Comprehension of the underlying mechanism of the anti-tumor action of MPA is imperative to facilitate its therapeutic potential as an anti-cancer agent. Herein, our reexamination of prior investigations has underscored a markedly elevated anti-proliferative efficacy of MPA against HNF4α-high cancer cells compared to HNF4α-low/negative cancer cells [43] (Supplementary Fig. S6). The inhibitory effects of MMF on GC cell migration were similarly observed in HNF4α-high AGS, NUGC-4, and KATOIII cells rather than HNF4α-low/negative OCUM-1 and HGC-27 cells (Figs. 2A, 2B and 8B–D and Supplementary Fig. S4B). Notably, HNF4α7 overexpression conferred MMF with the capability to suppress peritoneal metastasis of HGC-27 cells in nude mice (Fig. 8C, D). Hence, HNF4α may serve dual roles as both a therapeutic target and a biomarker for evaluating the therapeutic effectiveness of MMF across various cancer types, extending beyond advanced GC.

To be noted, MMF has been widely used in clinics for the prevention of acute graft rejection through inhibiting inosine monophosphate dehydrogenase (IMPDH), a rate-limiting enzyme in de novo synthesis of guanosine nucleotides; MPA has a more potent cytostatic effect on T- and B-lymphocytes due to their higher dependency on this pathway, as compared to other cell types [47]. For the application of MMF as an anti-tumor drug, a point to be considered is that the immunosuppressive activity of MPA may counteract its anti-tumor effect, if a particular tumor entity is immunogenic. Encouragingly, several clinical observations indicate that exposure to MMF is not linked to an elevated risk of cancer and may even be associated with a reduced risk of cancer in recipients of solid organ transplants [48, 49]. Additionally, MMF administration has been noted to ameliorate certain immune-related adverse events induced by immune checkpoint inhibitors in non-small cell lung cancer and advanced GC treatment [50, 51]. These observations underscore the promising potential of MPA as an anti-tumor agent, particularly when combined with immunotherapy, offering enhanced efficacy with diminished side effects.

In conclusion, our findings elucidate the molecular mechanisms underlying GC cell migration and metastasis, highlighting HNF4α as a potential therapeutic target (Fig. 8G). By leveraging an FDA-approved drug targeting HNF4α, we propose a promising avenue for inhibiting advanced GC. This discovery holds the potential to facilitate the development of prognostic tools and treatment strategies tailored to advanced GC.

## Materials and methods

### Bioinformatic analyses

The mRNA and clinical characteristic data of GC patients were acquired from the TCGA database through UCSC Xena (https://xenabrowser.net/datapages/) and the GEO database (https://www.ncbi.nlm.nih.gov/geo/). The expression relationships between HNF4α and other genes were assessed using two-sided Pearson’s product-moment correlation. Gene Set Enrichment Analysis (GSEA) was conducted using the R package (version 4.3.1) “clusterProfiler” (version 4.8.2). The R package “pheatmap” (version 1.0.12) was employed for generating gene expression heatmaps. “EnhancedVolcano” (version 1.18.0) was utilized to create volcano plots illustrating significant differential genes between HNF4α knockdown and control groups. “ggplot2” (version 3.4.2) was employed for visualizing gene expression correlation through scatter plots.

Single-cell RNA sequencing (scRNA-seq) data specific to GC were acquired from GSE163558 in the GEO database. Batch effects were eliminated using the “Harmony” R package (version 0.1.1). The single-cell dataset underwent processing with the “Seurat” R package (version 4.3.0). Visualization of the UMAP plot and the depiction of the expression pattern of HNF4α were accomplished using the functions “Dimplot” and “FeaturePlot” within the Seurat package.

For ChIP-seq analyses, we acquired HNF4α ChIP-seq data of GC cell lines and H3K4me1 ChIP-seq data of IM95 from GSE114018. Additionally, H3K4me3 and H3K27ac ChIP-seq data of the IM95 cell line were obtained from GSE75898. The “SRA toolkit” (version 2.8.0) was utilized to download SRA files from GEO or SRA at the National Center for Biotechnology Information (NCBI). Fastq-dump was employed to convert SRA files to FASTQ format, followed by trimming using Trim_galore (version 0.6.10). Sequence reads were mapped against the human reference genome hg19 using Bowtie2 (version 2.3.5.1). Subsequently, SAMtools (version 1.9) was applied to process BAM files, and PCR duplicates were removed using rmdup. MACS2 (version 2.2.9.1) was employed to call peaks for normalization, thereby identifying potential HNF4α binding sites. For visualization purposes, IGV (Integrative Genomics Viewer) was used to convert BAM files to TDF files.

### GC tissue microarray and immunohistochemical (IHC) staining

The gastric tissue microarrays (HStmA050Me01, Slice Number: M006 and M14, Shanghai Outdo Biotech Co.Ltd, Shanghai, China) were employed for IHC staining and then scored for statistical analysis. Each microarray included a total of 50 paraffin-embedded gastric tissue samples (including 17 primary lesions, 16 distant metastases, and 17 corresponding paracancerous tissues or normal tissues) derived from patients diagnosed with metastatic GC. The study was conducted in accordance with the Declaration of Helsinki, and approved by the Internal Ethical Review Committee of Xinchao Biotechnology (Shanghai, China) (Ethical Number: SHYJS-CP-1501014, approval date: 9 January 2015).

For IHC staining, the gastric tissue microarrays were deparaffinized and rehydrated, and then treated with citrate antigen retrieval solution to enhance antigen exposure. Following this, the slides were incubated with primary antibodies at 4 °C overnight. To quench endogenous peroxidase activity, probably interfering with the IHC staining process, the UltraSensitive^TM^ SP (Mouse/Rabbit) IHC Kit (Cat. #Kit-9710, MXB Biotechnologies, Fuzhou, China) was applied. The subsequent steps of secondary antibody incubation and 3,3’-diaminobenzidine (DAB) development were executed in strict accordance with the manufacturer’s instructions. After the DAB reaction, the sections were counterstained with hematoxylin to provide a contrasting blue color to the cell nuclei. The slides then underwent a series of dehydration steps using graded alcohols, followed by clearing with xylene to remove any residual solvent, and finally sealed under coverslips for examination. The slides were digitized using a Leica Aperio Versa 200 scanner, yielding high-content, high-resolution digital images.

### Cell culture

Human embryonic kidney cell lines 293 T and human GC cell lines HGC-27, NCI-N87, and KATO-III were obtained from the Cell Bank, Type Culture Collection, Chinese Academy of Sciences, Shanghai, China. Human GC cell lines AGS were kindly provided by Songlin Shi (Xiamen University, Xiamen, Fujian, China). Human GC cell lines NUGC-4 and OCUM-1 were obtained from MeisenCTCC (Meisen Chinese Tissue Culture Collections), Zhejiang, China. All cell lines were identified by STR profiling and confirmed to be mycoplasma negative by the source. Upon receipt, the cells were expanded and subsequently cryopreserved in liquid nitrogen. Thawing of the storage vials was performed prior to experiments, and the cells were utilized within a period of less than two months. Rigorous screenings were consistently conducted to reaffirm the absence of mycoplasma in all cell lines.

Specifically, 293 T and OCUM-1 cells were cultured in Dulbecco’s modified Eagle’s medium (DMEM, Gibco, Grand Island, NY, USA), while HGC-27, AGS, and NUGC-4 cells were maintained in RPMI 1640 medium (Gibco, Grand Island, NY, USA). NCI-N87 cells were cultured in RPMI 1640 medium supplemented with 1% glutamax (Invitrogen, Carlsbad, CA, USA) and 1 mM sodium PA (Invitrogen, Carlsbad, CA, USA). KATO-III cells were cultured in Iscove’s modified Dulbecco’s medium (IMDM, Gibco, Grand Island, NY, USA). All culture media were supplemented with 10% fetal bovine serum (FBS, HyClone, Logan, UT, USA), 100 U/mL penicillin, and 100 μg/mL streptomycin (Life Technologies, Carlsbad, CA, USA). The cells were incubated at 37 °C in a humidified incubator containing 5% CO_2_.

### Antibodies and reagents

Mouse anti-HNF4α antibodies, including H1415 (pan-specific for P1/P2-HNF4α isoforms; Cat. #417700; used at 1:1000 for WB across all figures, 1:200 for immunohistochemistry (IHC), 1:200 for IF, and 1:200 for chromatin immunoprecipitation (ChIP)), K9218 (P1-HNF4α-specific; Cat. #MA1-199; 1:1000 for WB in Fig. 2A and 1:200 for IHC), and H6939 (P2-HNF4α-specific; Cat. #417800; 1:1000 for WB in Fig. 2A and 1:200 for IHC), mouse IgG Isotype Control (Cat. #02-6502, 1:200 for ChIP), and goat anti-mouse Alexa Fluor 488 secondary antibody (Cat. #A-10680) were purchased from Thermo Scientific, Waltham, MA, USA. Mouse anti-β-actin antibody (Cat. #A3854, 1:10000 for WB) was purchased from Sigma-Aldrich, St. Louis, MO, USA. Goat anti-Lamin B (Cat. #sc-6216,1:5000 for WB) antibody was purchased from Santa Cruz Biotechnology, Santa Cruz, CA, USA. Mouse anti-β-tubulin antibody (Cat. #EP1331Y, 1:5000 for WB) was purchased from Abcam, Cambridge, MA, USA. Rabbit anti-HKDC1 antibody (Cat. #25874-1-AP, 1:1000 for WB) was obtained from Proteintech, Wuhan, China. Rhodamine Phalloidin (Cat. #PHDR1) was obtained from Cytoskeleton, Cambridge, MA, USA.

MMF (Cat. #HY-B0199) was purchased from Med Chem Express, Shanghai, China. Matrigel (Cat. #354234), ultra-low cluster, 96 well round bottom, low ament polystyrene, non-pyrogenic (Cat. #67) was purchased from Corning, Corning, New York, USA. Glass Bottom Cell Culture Dishes (Cat. # 801002) were purchased from Cell-Nest, Wuxi, Jiangsu, China.

### Plasmids

Human HNF4α7 cDNA (kindly provided by Professor Frances M Sladek, University of California, Riverside, CA, USA) and human HKDC1 cDNA (obtained by reverse transcription using RNA from AGS cells) were cloned into vectors as needed, such as pLV-sf-GFP (2A) Puro and pGEX-4T-1. The predicted HNF4α binding region on the *HKDC1* promoter was inserted into the pGL6-TA vector.

The HNF4α7(ΔDBD, the deletion of the DNA binding domain spanned amino acids 38 to 113) plasmid was constructed through a process of homologous recombination. Primers were specifically designed to match a 15-base sequence upstream and a sequence with a 60 °C melting temperature downstream of the deletion fragment, both derived from the HNF4α7 plasmid. Following PCR amplification, the products were treated with DpnΙ to digest any remaining methylated parental DNA and subsequently purified through gel electrophoresis. The purified PCR products were then subjected to ligation mediated by a homologous recombinase. The ligated products were transformed into competent cells, and the resulting plasmids were isolated and sequenced to confirm the successful incorporation of the desired deletion.

The specific shRNA sequences targeting human HNF4α were: 5′-GATCAGCACTCGAAGGTCAA-3′ (for shHNF4α-1) and 5′-GCGTGGTGGACAAAGACAA-3′ (for shHNF4α-2). The shRNA sequence targeting human HKDC1 was 5′-TCATGAAGACCAAAGATTTAA-3′. The shRNA control (scramble) sequence employed was 5′-CAACCCGCTCCAAGGAATCG-3′. Oligonucleotides (Invitrogen, Guangzhou, China) were annealed and subsequently inserted into the pLKO vector.

### Lentivirus-mediated stable knockdown or overexpression

Lentiviruses were produced through the transfection of 293T cells with the lentiviral vector (pLKO or pLV) along with packaging plasmids (pMDLg-pRRE, pRSV-REV, and pCMV-VSV-G) utilizing polyethylenimine (PEI, Cat. #23966, Polysciences, Warrington, PA). Viral supernatants were harvested 48 h post-transfection and filtered through 0.45 μm filters (Millipore, Billerica, MA, USA). Freshly plated AGS and HGC-27 cells were infected with the packaged lentivirus and subjected to puromycin selection (2 μg/mL).

### WB

Cells were lysed in cold ELB buffer (50 mM Tris, pH 7.6, 140 mM NaCl, 100 mM NaF, 0.5% NP-40, 2 mM NaVO_3_, 5 mM β-Glycerol phosphate, and 1 mM PMSF) and subsequently centrifuged at 13,200×*g*, 4 °C for 30 min. The resulting supernatant was collected, and the protein concentration was determined using a BCA assay kit (Bio-Rad, Hercules, CA, USA). The cell lysate was mixed with an equal volume of 2 × loading buffer, boiled at 100 °C for 15 min, and then subjected to sodium dodecyl sulfate-polyacrylamide gel electrophoresis (SDS-PAGE) and transferred to a polyvinylidene fluoride (PVDF) membrane. After blocking with 5% non-fat dry milk in TBS-T buffer (20 mM Tris, pH 7.5, 100 mM NaCl, and 0.1% Tween-20) for 1 h at room temperature, the membranes were incubated with the primary antibody overnight at 4 °C. Following three washes with TBS-T (5 min each), the membranes were incubated with the secondary antibody for 3 h at room temperature, followed by three washes with TBS-T (10 min each). The immune complexes were visualized using an ECL kit (Millipore, Bedford, MD, USA).

### Total RNA extraction and real-time PCR

Total RNA was extracted using the RNAiso Plus reagent following the manufacturer’s instructions (Takara, Dalian, China). Subsequently, cDNA was synthesized by reverse transcription using 1 μg of total RNA and Primescript™ RT Master Mix (Takara, Dalian, China). Real-time PCR was conducted employing SYBR Green I fluorescent dye (SYBR® Premix Ex Taq^TM^ II, TaKaRa, Dalian, China) and the StepOnePlus^TM^ real-time PCR system (Applied Biosystems, Australia). GAPDH was utilized as an internal control for normalization.

The primer sequences used for real-time PCR were listed as follows:

*P1-HNF4α* 5’- ATGCGACTCTCCAAAACCCT-3’ (forward)

5’- GTGTCATTGCCCATCGTCAA-3’ (reverse)

*P2-HNF4α* 5’- AGTGGAGAGTTCTTACGACAC-3’ (forward)

5’- CAGGAGTACATGTGGTTCTTC-3’ (reverse)

*P1/P2-HNF4α* 5’-CGACATTCGGGCGAAGAAGA-3’ (forward)

5’-TGTACTTGGCCCACTCAACG-3’ (reverse)

*HKDC1* 5’-AGCATGTCTGTACCATCGTCTCCTT-3’ (forward)

5’-GCCGTTCCACCTTCTTGTTCTCC-3’ (reverse)

*GAPDH* 5’-CACCATCTTCCAGGAGCGAG-3’ (forward)

5’-GACTCCACGACGTACTCAGC-3’ (reverse)

### Wound healing assay

Cells were seeded and cultured in a 6-well plate. When the cell confluence reached 90%, a gentle and slow scratch was carefully made across the center of the dishes using a new 20 μL pipette tip. Following the scratch, cells were washed three times with PBS to eliminate cellular debris and then cultured in fresh medium containing 1% serum. Images were captured at the indicated time point using an Olympus inverted microscope (Tokyo, Japan) at ×20 magnification. The gap distance was quantitatively assessed. The relative migration distance of cells was determined by dividing the non-filled area by the initial distance. The data were presented as mean ± SEM.

### Transwell migration and invasion assay

Cells (8 × 10^4^) were suspended in serum-free medium and seeded onto the upper chamber of Boyden chambers with an 8.0-μm pore size Boyden chambers (Corning, New York, USA) (for AGS and HGC-27 cells) or 12.0-μm pore size chambers (JET BIOFIL, Guangzhou, China) (for NUGC-4, KATO-III, and OCUM-1 cells), which were either coated with Matrigel (for invasion assays) or left uncoated (for migration assays). The lower chamber was filled with complete medium to serve as a chemoattractant. Following a 24-h incubation in a standard cell culture incubator, the chambers were carefully removed, and cells that did not migrate or invade through the membrane were gently wiped off using a cotton swab.

Subsequently, the chambers were fixed with 4% paraformaldehyde at room temperature for 10 min and then stained with 0.1% crystal violet for an additional 10 min. Random microscopic fields were selected for photography, and cell counts were conducted.

### Single-cell trajectory tracking

One of the AGS shCtrl and shHNF4α cell sublines (or one of the HGC-27 pLV-Vector and pLV-HNF4α7 cell sublines) was labeled with CellTracker™ CM-Dil Dye (Cat. #C7001, Thermo Scientific) for 30 min. A total of 2 × 10^4^ labeled cells, along with an equal number of unlabeled cells, were co-cultured and seeded onto a Glass Bottom Cell Culture Dish (Cat. #801002, Cell-Nest, Wuxi, Jiangsu, China). The dish was then placed in an incubator set at 37 °C in a humidified atmosphere with 5% CO_2_ for cultivation until cells adhered to the surface and fully recovered. Time-lapse imaging was performed at 5-min intervals using a FV1000 confocal laser-scanning microscope at a magnification of ×20. The spatial distribution of cells (*n* ≥ 25 per group) was subsequently analyzed using ImageJ software (National Institutes of Health, Bethesda, MD, USA).

### 3D tumor spheroid migration and invasion assay

Cells (5 × 10^3^) were suspended in 200 μL of complete medium, added to wells of ultra-low attachment (ULA) 96-well round-bottom plates, and centrifuged at 1000×*g* for 10 min. The plates were then placed in an incubator (37 °C, 5% CO_2_, 95% humidity). Forty-eight hours later, successful tumor spheroid formation was visually confirmed. For the migration assay, 500 μL of complete medium was added to wells in a 24-well plate. The tumor spheroid was carefully transferred to the center of the well and cultured for 3 h. Subsequently, the complete medium was replaced with serum-free medium (designated as 0 h), and photographs of the tumor spheroids were taken at the indicated time points to analyze the migration ability of the tumor cells in a 3D environment. In the invasion assay, after discarding the complete medium in the 24-well plate, 100 μL of diluted Matrigel (the Matrigel was diluted with serum-free medium at a ratio of 1:17) was gently added, minimizing disturbance to the spheroids. Once the Matrigel solidified, 500 μL of complete medium was gently added on top of the Matrigel (designated as 0 h). Similar to the migration assay, photographs of the tumor spheroids were taken at the indicated time points to analyze their invasion ability.

### MTT assay

Cells were plated in 96-well plates at a density of 4 × 10^3^ per well. At specified time intervals, 20 μL of MTT (Sigma-Aldrich, St. Louis, MO, USA, 5 mg/mL) was introduced into each well and incubated for 4 h. Following this, the medium was substituted with 200 μL of DMSO to dissolve the formazan crystals, and the quantification of viable cells was conducted by measuring the absorbance at 570 nm (Abs) using a spectrophotometer.

### IF assay

Cells cultured on cover glasses were fixed with 4% paraformaldehyde for 10 min at room temperature and then permeabilized with 0.2% Triton X-100 in PBS for 10 min on ice. Following this, the coverslips were blocked with 5% BSA in PBS for 1 h at room temperature. Subsequently, they were incubated with primary antibodies overnight at 4 °C. Afterward, coverslips were washed three times with washing buffer (1.5% BSA, 0.02% Triton X-100 in PBS) and incubated with Alexa Fluor 488-conjugated secondary antibodies for 3 h at room temperature. Following another wash with washing buffer, coverslips were sealed with mounting medium containing DAPI (Cat. #ab104139, Abcam, Cambridge, MA, USA). The samples were visualized and photographed using an FV1000 confocal laser-scanning microscope.

### Extraction of nuclear and cytoplasmic fractions

Cells were harvested and suspended in 200 μL Buffer A (10 mM KCl, 0.1 mM EDTA, 10 mM HEPES, pH 7.9, 0.1 mM EGTA, 2 mM NaVO_3_, 5 mM β-Glycerol phosphate, 0.15% NP-40, and 1 mM PMSF) and kept on ice for approximately 15 min. Subsequently, the homogenates were centrifuged at 13,200×*g*, 4 °C for 2 min, and the resulting supernatants were collected to obtain the cytosolic fraction. For the nuclear fraction, the pellets were washed five times with Buffer A solution and centrifuged at 10,000×*g*, 4 °C for 1 min after each wash. The supernatants were discarded, and the pellets were resuspended in 100 μL Buffer B solution (400 mM NaCl, 20 mM HEPES, pH 7.9, 1 mM EGTA, 1 mM EDTA, 0.5% NP-40, 5 mM β-Glycerol phosphate, 2 mM NaVO_3_, and 1 mM PMSF) followed by lysis with an ultrasonic field. The lysates were then centrifuged at 13,200×*g*, 4 °C for 30 min, and the resulting supernatant was collected as the nuclear fraction.

### ATP assay

The concentration of ATP in cells was detected using the ATP Assay Kit (Beyotime, Cat. #S0026), based on the manufacturer’s instructions. In brief, cells were lysed, and the supernatant was collected after being centrifuged at 12,000×*g*, 4 °C for 5 min. To each well of a 96-well microplate, 100 μL of the test solution was aliquoted, followed by the addition of 20 μl of the supernatant. The luminescence, indicative of ATP presence, was then measured using a luminometer. The relative ATP values were normalized by dividing the luminescence values by the corresponding protein concentrations.

### Lactate concentration measurement

The lactate concentration in cells was analyzed using the L-Lactic Acid (LA) Colorimetric Assay Kit (Elabscience, Cat. #E-BC-K044-M). In brief, cells were resuspended in PBS and sonicated on ice. Lysates were centrifuged at 10,000×*g*, 4 °C for 10 min. Then the supernatant, along with the working solution, was added to a 96-well microplate and incubated for 10 min at 37 °C to allow for color development. The OD value of the samples was measured at 530 nm using a microplate reader. The relative lactate levels were calculated by dividing the measured OD_530_ values by the respective protein concentrations.

### Detection of cellular ROS levels

To assess the levels of ROS within cells, we utilized the ROS Assay Kit (Beyotime, Catalog #S0033S). Initially, cells were seeded and allowed to adhere overnight. Subsequently, the cells were treated with the fluorescent probe DCFH-DA and incubated for 20 min at 37 °C to facilitate intracellular uptake. Following the incubation, cells were thoroughly washed three times with a serum-free cell culture medium to eliminate any uninternalized DCFH-DA. The intracellular ROS levels were then visualized using a fluorescence microscope. The relative fluorescence intensity, indicative of ROS levels, was quantified with the aid of ImageJ software.

### Untargeted or targeted metabolomics analysis

For cell culture samples, metabolites were extracted from cell lysates using a pre-cooled solvent mixture of methanol, acetonitrile, and water (2:2:1, *v*/*v*/*v*). The cells were lysed by ultrasonication on ice, and the resulting lysate was incubated at –20 °C for 1 h to precipitate proteins. After centrifugation at 13,000 rpm and 4 °C for 15 min, the supernatant was collected and directly injected into the LC–MS system for metabolomic analysis using U-¹³C-fructose as an internal standard.

For tumor tissues obtained from mice, freshly resected samples were immediately snap-frozen in liquid nitrogen. Approximately 40 mg of tissue was sectioned and homogenized three times in 200 μL of ice-cold ultrapure water. Then, 800 μL of extraction solvent (methanol:acetonitrile, 1:1, *v*/*v*) was added, followed by vortexing for 30 s and ultrasonication in an ice bath for 10 min. The mixture was incubated at –20 °C for 1 h to enhance protein precipitation. After centrifugation under the same conditions as used for the cell samples, 800 μL of supernatant was collected, concentrated to dryness via low-temperature vacuum centrifugation, and the resulting lyophilized powder was reconstituted for LC–MS analysis.

### Dual-luciferase reporter assay

Luciferase assays were performed using the Dual-Glo^TM^ Luciferase Assay System (Promega, Madison, WI, USA), and luminescence was quantified on a GloMax 20/20 Luminometer (Promega) following the manufacturer’s guidelines. The luciferase activity was determined by normalizing Firefly luciferase activity to Renilla luciferase activity.

### Electrophoretic mobility shift assay (EMSA)

To assess the direct binding of HNF4α to the HKDC1 promoter, recombinant GST-HNF4α was initially generated. GST, GST-HNF4α, or GST-HNF4α(△DBD) was expressed in E. coli BL21(DE3) strain through isopropyl-β-thiogalactoside (IPTG) induction (0.1 mM, 12 h, 20 °C), followed by purification using glutathione-Sepharose (Cat. #1057865, QIAGEN, Hilden, Germany) and elution with elution buffer (50 mM Tris, 10 mM glutathione, pH 8.0). The resulting purified protein was dialyzed against PBS, and its concentration was determined using the BCA Protein Assay Kit.

Double-stranded 5’biotin-labeled and unlabeled oligonucleotides (Sangon Biotechnology, Shanghai, China) containing the predicted HNF4α binding site on the HKDC1 promoter (S3: +4323 ~ +4381 bp) served as probes. For each reaction, 1 μg of purified protein, 20 fmol of labeled DNA, and 4 pmol of unlabeled DNA were utilized. Protein-DNA binding reactions were conducted using the LightShift™ Chemiluminescent EMSA Kit (Cat. #20148, Thermo Fisher Scientific, Waltham, MA, USA) in accordance with the provided instructions. Subsequent to completion of the reaction, the mixtures were loaded onto a 6% polyacrylamide gel, subjected to electrophoresis, and then transferred to a nylon membrane (Hybond-N, Amersham Pharmacia Biotech, Little Chalfont, UK), which underwent UV crosslinking for 15 min. Visualization of the biotin-labeled DNA was accomplished using the Chemiluminescent Nucleic Acid Detection Module within this kit.

### ChIP

Cells were subjected to cross-linking with 1% formaldehyde for 10 min at room temperature, and the reaction was quenched by the addition of glycine to a final concentration of 0.25 M. Following cell harvesting using a scraper, the cellular material was resuspended in 1 mL of lysis buffer (50 mM HEPES, pH 7.5, 140 mM NaCl, 1 mM EDTA, 1% Triton X-100, 0.1% SDS, 0.1% sodium deoxycholate) and sonicated to achieve DNA fragments ranging from 200 to 2000 bp. After centrifugation at 12,000 g and 4 °C for 10 min, 1/10 of the supernatant was reserved as the input control, while the remaining supernatant underwent pre-clearing with 30 μL Protein A/G Plus Agarose (pre-treated with DNA from salmon testes for 30 min at room temperature) to mitigate non-specific binding. Subsequently, the pre-cleared lysates were incubated with 30 μL pre-treated Protein A/G Plus Agarose and 2 μg HNF4α antibody (Cat. #H1415, Thermo Scientific) or the corresponding mouse IgG isotype control (Cat. #02-6502, Thermo Scientific) overnight at 4 °C. The agarose was then subjected to washing steps, including once with low salt buffer (20 mM Tris-HCl, pH 8.0, 150 mM NaCl, 0.1% SDS, 1% Triton X-100, 2 mM EDTA), high salt buffer (20 mM Tris-HCl, pH 8.0, 0.5 M NaCl, 0.1% SDS, 1% Triton X-100, 2 mM EDTA), LiCl buffer, and twice with TE buffer. Protein-DNA complexes were extracted using 500 μL elution buffer (1% SDS, 0.1 M NaHCO3, 0.5 mg/mL RNase A) with rotation at room temperature for 15 min. Crosslinks were reversed by heating at 65 °C for 4 h, followed by the addition of 10 μL 0.5 M EDTA and 1 μL proteinase K (20 mg/mL), and incubation at 55 °C for 1 h. DNA was purified through phenol/chloroform extraction, and the resulting pellets were dissolved in 50 μL double distilled water for subsequent semi-quantitative PCR and real-time PCR. The primer sequences used are provided below:

Human HKDC1 promoter, 5’-ATTACAGGCGTGAGCCACTG-3’ (S3, forward)

5’-AGCAGCCCGTTTCACTTAGA-3’ (S3, reverse).

### Peritoneal metastasis assay

All animal experiments were performed in accordance with a protocol approved by the Animal Care and Use Committee of Xiamen University. Female nude mice (BALB/c, 15–17 g, 5 weeks old) were obtained from the SLAC Laboratory Animal Center (Shanghai, China) and maintained under specific pathogen-free conditions in the Laboratory Animal Centre of Xiamen University. A total of 1 × 10^7^ cells stably expressed luciferase (HGC-27 pLV-Vector or pLV-HNF4α7 cell subline, or AGS shCtrl or shHKDC1 cell subline) were suspended in 200 μL of RPMI-1640 medium and subsequently injected into the peritoneal cavity of each mouse (*n* = 6 or 7 per group as indicated). Thirty days (for HGC-27 cell sublines) or forty days (for AGS cell sublines) later, tumor peritoneal cavity metastasis was measured using a Caliper IVIS Lumina II Kinetic system (Caliper Life Science, USA) 5 min after 15 μg of luciferin (Cat. #E1605, Promega, 15 mg/mL in phosphate-buffered saline (PBS)) was intraperitoneally injected.

To detect the effect of MMF on GC peritoneal metastasis, one of the HGC-27 pLV-Vector and pLV-HNF4α7 cell sublines (all cell sublines stably expressing luciferase, 1 × 10^7^ cells per mouse) was injected into the nude mice as described above. Ten days later, the mice were randomly divided into two groups (*n* = 7 per group), given vehicle (0.9% NaCl) or MMF (40 mg/kg) once daily for 26 days by oral administration.

### Real‑time cell metabolism assay

The Seahorse XF96 extracellular flux analyzer (Seahorse Bioscience, North Billerica, MA, USA) was employed for real-time analysis of the OCR and the ECAR. Briefly, cells (2.5 × 10^4^/well) were seeded on XF96 Cell Culture Microplates (Cat# 102416-100, Seahorse Bioscience, Agilent, CA, USA). Eighteen hours later, the culture medium of adherent cells was replaced with sterile Seahorse XF base media (Cat# 102353-100 for OCR and Cat# 103335-100 for ECAR) supplemented with 10 mM D-glucose, 2 mM L-glutamine, and 1 mM sodium PA (pH 7.4). Real-time OCR and ECAR measurements were conducted following the manufacturer’s instructions.

Mitochondrial respiration was assessed in XF assay media under basal conditions and in response to 1 μM oligomycin, 1.5 μM fluorocarbonyl cyanide phenylhydrazone (FCCP), 1 μM rotenone, and 1 μM antimycin A (R/A). Glycolysis capacity was determined under basal conditions and in response to 10 mM glucose, 1 μM oligomycin, and 50 mM 2-deoxyglucose (2-DG). Both OCR and ECAR values were normalized to the total protein content. This methodology provided a dynamic and real-time assessment of cell metabolism in GC cells under various conditions.

### Statistical analysis

All the data were processed as means ± SEM, and the statistical significance was determined by two-tailed unpaired Student’s *t*-test, one-way ANOVA, or two-way ANOVA using the GraphPad Prism 8 (GraphPad Software).

## Supplementary information

Supplementary Information

Full uncropped western blots

## Data Availability

The datasets generated and/or analyzed during the current study are available from the corresponding author upon reasonable request.
