# Supplementary material for: HNF4α-HKDC1 axis orchestrates a metabolic rewiring to promote migration and metastasis in advanced gastric cancer
Source: Cell Death Dis. 2026 Mar 23;17(1):347. doi: 10.1038/s41419-026-08627-y (PMC13039212; doi:10.1038/s41419-026-08627-y)
Supplement: Supplementary file 1 — Supplementary Information [file 41419_2026_8627_MOESM1_ESM.docx]

Supplementary data for

**HNF4α-HKDC1 axis orchestrates a metabolic rewiring to promote migration and metastasis in advanced gastric cancer**

Xiaolin Xu, Han Wu, Jin Shang, Yating Wang, Yifan Yang, Tianying Cai, Lu Chen, Xuechun Xu, Chenyu Zhang, Wenqing Zhang, Daxuan Wang, Mingqing Zhang, Yan-yan Zhan

**Supplementary Figures**


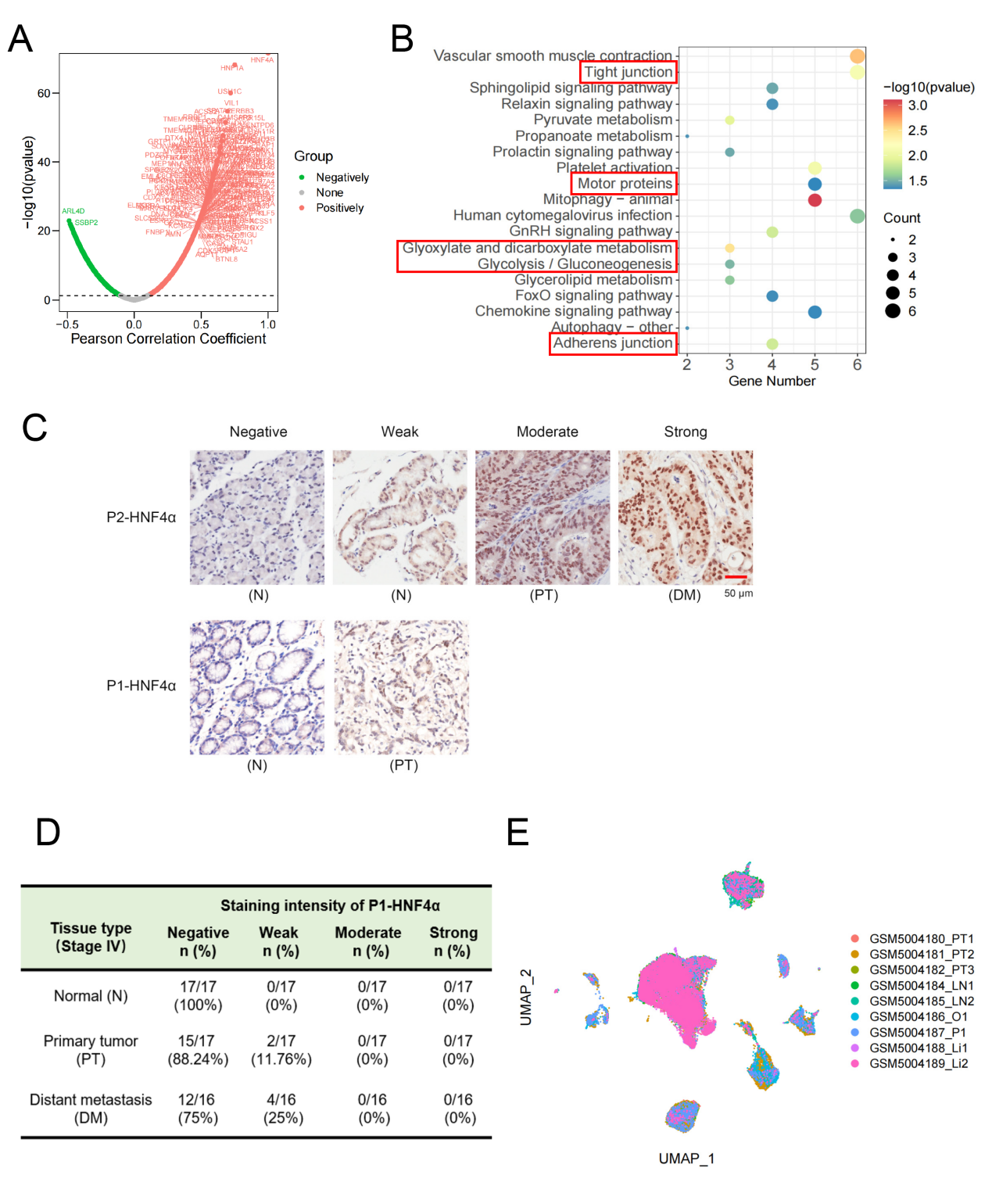


**Supplementary Figure S1. P2-HNF4α but not P1-HNF4α was highly expressed in human metastatic GC samples.** (**A**) Volcano plot displaying the top 200 genes with a significant Pearson correlation to HNF4α in the GC-related dataset from TCGA database. (**B**) KEGG enrichment analysis of genes associated with HNF4α from (A).

**(C)** Representative images of the criteria (negative, weak, moderate, and strong) used for grading the IHC results of P2-HNF4α or P1-HNF4α expression in paracancerous or normal gastric tissues (N), primary tumors (PT), or distant metastases (DM). (a) Negative expression of P2-HNF4α or P1-HNF4α. (b) Weak positive expression of P2-HNF4α or P1-HNF4α. (c) Moderately positive expression of P2-HNF4α. (d) Strongly positive expression of P2-HNF4α. Scale bar: 50 μm. (**D**) Statistical representation of P1-HNF4α expression levels (immunohistochemical staining intensity) in clinical specimens of metastatic GC patients, including paracancerous or normal gastric tissues (N, n = 17), primary tumors (PT, n = 17), and distant metastases (DM, n = 16). (**E**) Uniform Manifold Approximation and Projection (UMAP) representation of nine gastric cancer samples (including both primary and metastatic samples). The scRNA-seq data for gastric cancer were obtained from GSE163558 in the GEO database.


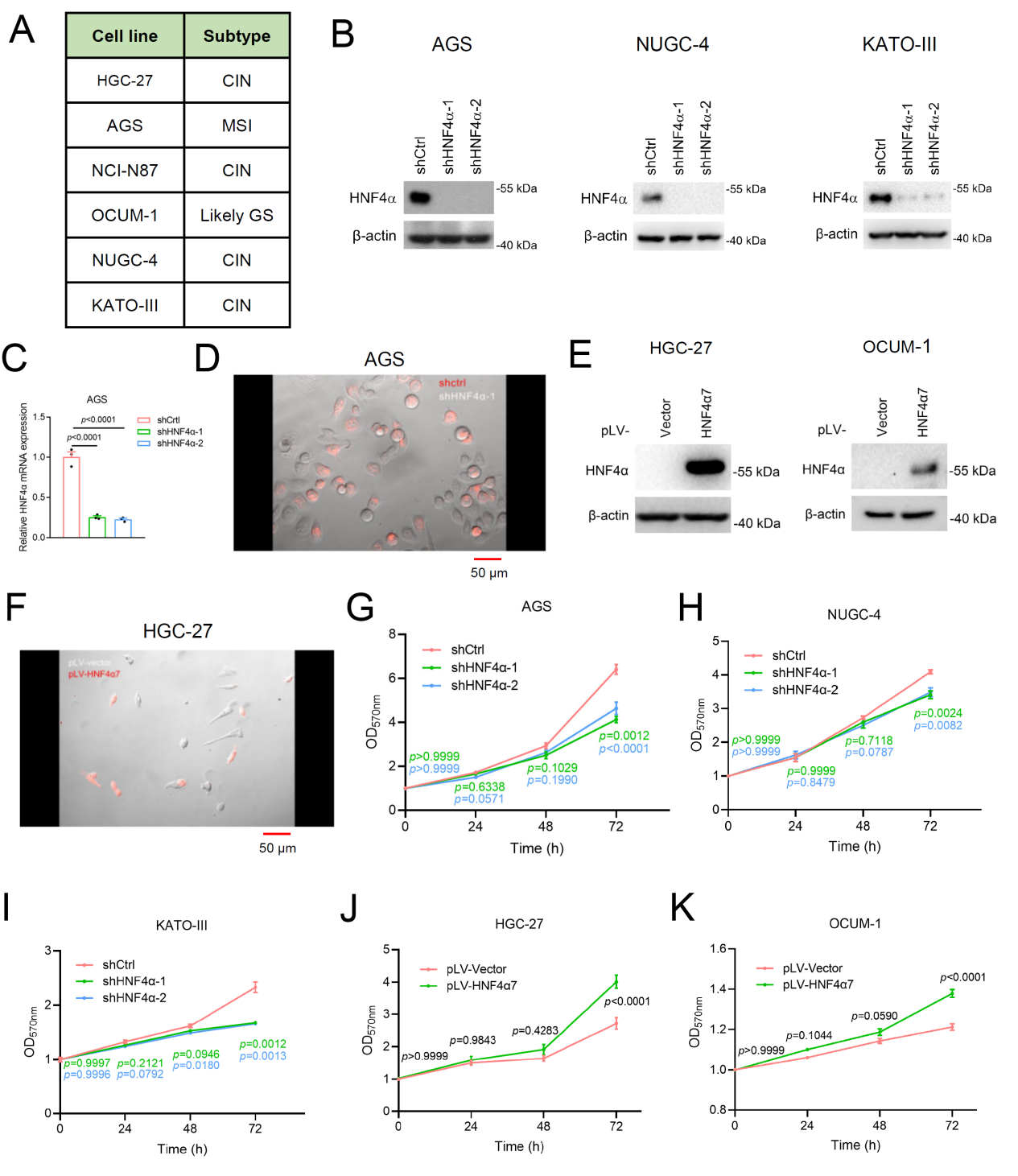


**Supplementary Figure S2. P2-HNF4α promoted the migration and invasion of GC cells.** (**A**) Subtype information of 6 GC cell lines. (**B, C**) Interference effect of shRNA against HNF4α in AGS, NUGC-4, and KATO-III cells was examined by western blot (B) and real-time PCR (C). (**D**) A snapshot of the single-cell trajectory tracking assay comparing shCtrl and shHNF4α AGS cells was depicted in the screen capture. The shCtrl AGS cell subline (but not the shHNF4α subline) was labeled with CellTracker™ CM-Dil Dye. (**E**) The expression levels of transfected HNF4α7 in HGC-27 and OCUM-1 cells were examined by western blot with anti-HNF4α (H1415) antibody. (**F**) A screen capture illustrating the single-cell trajectory tracking assay for pLV-Vector and pLV-HNF4α7 sublines of HGC-27 cells was presented. The pLV-HNF4α7 HGC-27 cell subline, instead of the pLV-Vector subline, was labeled with CellTracker™ CM-Dil Dye. (**G–K**) The impact of HNF4α knockdown or overexpression on the growth or survival of AGS, NUGC-4, and KATO-III, HGC-27 and OCUM-1 cells was evaluated using the MTT assay. Data were presented as the mean ± SEM. The difference significance was analyzed by one-way ANOVA (**C, G–I**) or unpaired two-tailed Student’s *t*-test (**J, K**).

**Supplementary Figure S3. HKDC1 was the downstream target for HNF4α to promote GC metastasis.** **(A-F)** The efficacy of HKDC1 knockdown in AGS cells or HKDC1 overexpression in HGC-27 cells and their respective effects on GC cell migration, growth or survival was examined by real-time PCR (A, B), wound healing assay (C, D) and MTT assays (E, F). Representative images (C, D, left panel, scale bar: 100 μm) and quantification of wound distance (C, D, right panel) were shown. N = 4 (for A, B), 12 (for C), 8 (for D) or 5 (for E, F) biologically independent samples. (**G**) HKDC1 was overexpressed in AGS cells with HNF4α knockdown. The efficacy of HNF4α knockdown and HKDC1 overexpression was assessed through real-time PCR analysis. (**H**) HNF4α7 was overexpressed in control and HKDC1-knockdown HGC-27 cells. The efficacy of HNF4α overexpression and HKDC1 knockdown was evaluated through real-time PCR analysis. (**I)** The effect of HNF4α knockdown on ROS levels was rescued by HKDC1 overexpression in AGS cells. Representative images were shown (scale bar: 100 μm). Data were presented as the mean ± SEM. The difference significance was analyzed by unpaired two-tailed Student’s *t*-test (A-F), one-way ANOVA (G) or two-way ANOVA (H).


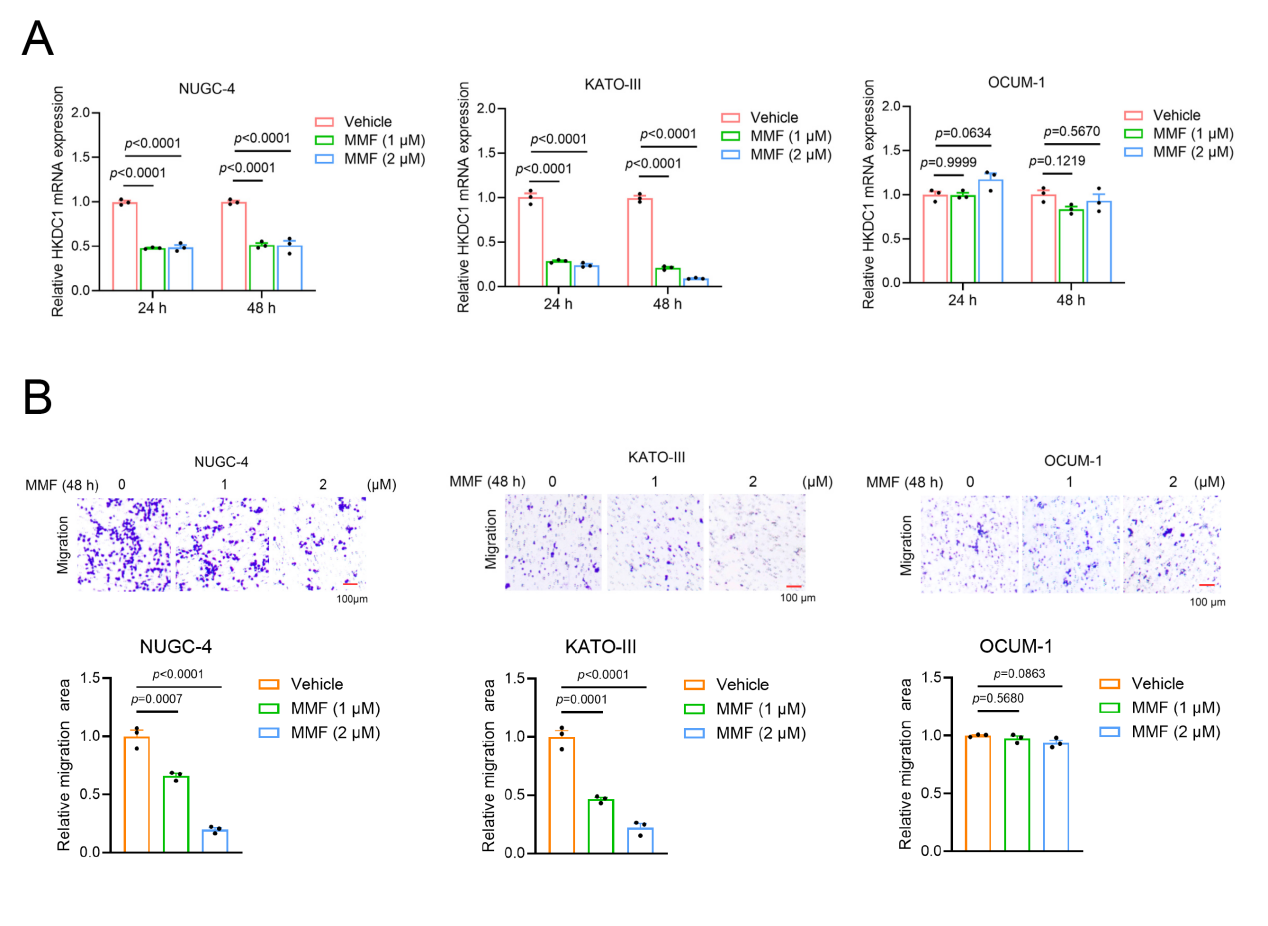


**Supplementary Figure S4.** **The suppressive effects of MMF on the mRNA expression level of HKDC1 and the migration of NUGC-4 and KATO-III but not OCUM-1 cells was detected by real-time PCR (A) and transwell migration assay (B).** Representative images (B, upper panel) and and relative migration area (B, lower panel) were shown (n = 3 biologically independent samples). Scale bar: 100 μm. Data were presented as the mean ± SEM. The significance of differences was analyzed by one-way ANOVA.


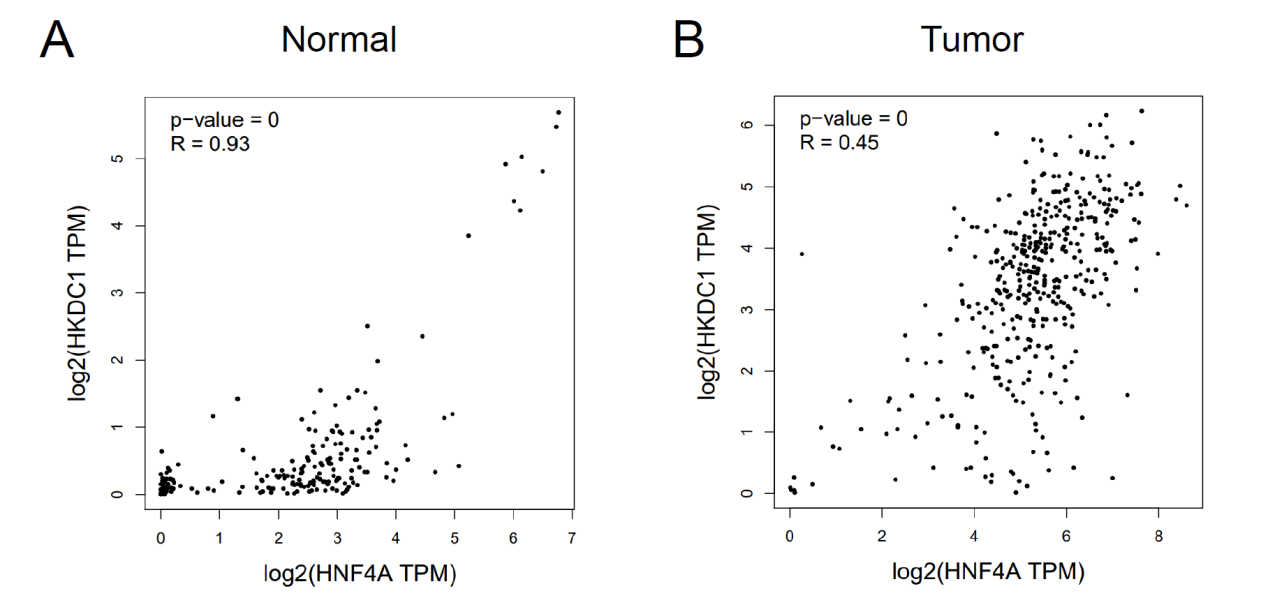


**Supplementary Figure S5.** (**A, B**) **The correlation between HNF4α and HKDC1 expression was observed in normal gastric samples (A) and GC samples (B) using GEPIA (http://gepia.cancer-pku.cn/).**


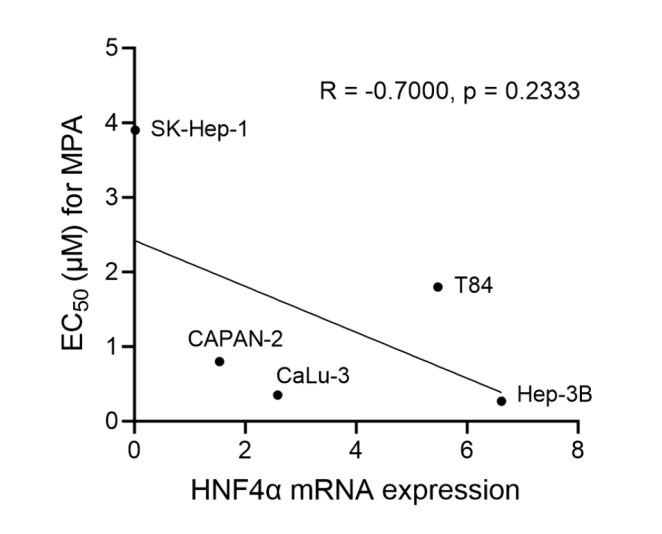


**Supplementary Figure S6. The correlation between the expression of HNF4α and the anti-tumor efficacy of mycophenolic acid (MPA) in cancer cells.** The correlation between HNF4α mRNA expression and the EC_50_ value of MPA. The EC_50_ values signified the concentration of MPA necessary to inhibit the growth of specified cells by 50% compared to the growth observed with the vehicle control. The HNF4α mRNA expression levels across diverse cancer cell lines were extracted from the CCLE database.
