# Supplementary material for: HNF4α-HKDC1 axis orchestrates a metabolic rewiring to promote migration and metastasis in advanced gastric cancer
Source: Cell Death Dis. 2026 Mar 23;17(1):347. doi: 10.1038/s41419-026-08627-y (PMC13039212; doi:10.1038/s41419-026-08627-y)
Supplement: Supplementary file 2 — Full uncropped western blots [file 41419_2026_8627_MOESM2_ESM.docx]

**Figure 2A**

P1/P2-HNF4α


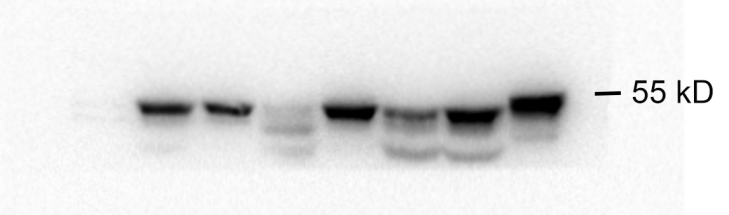


P1-HNF4α


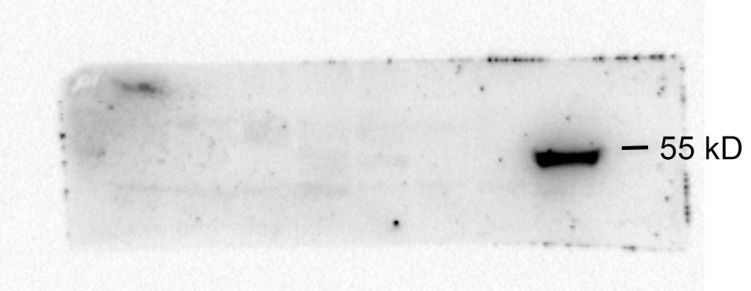


P2-HNF4α


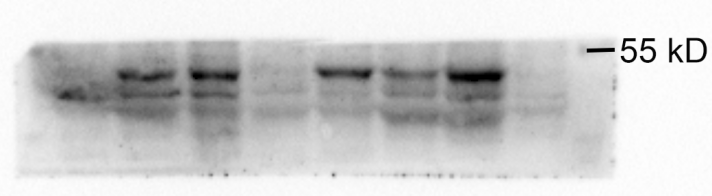


β-actin


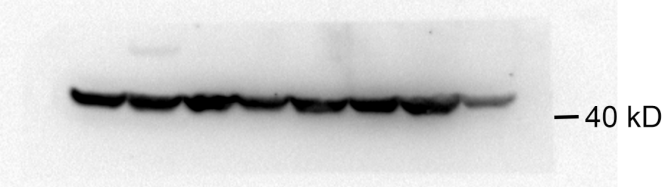


**Figure 3B**


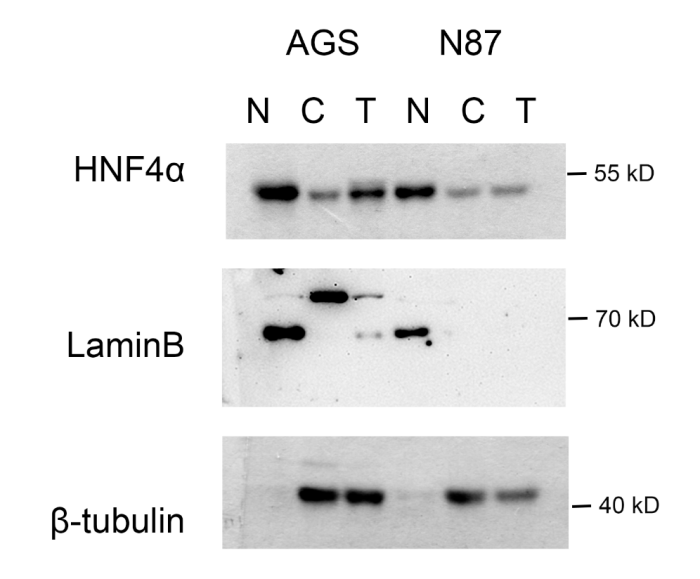


**Figure 3C**

HNF4α


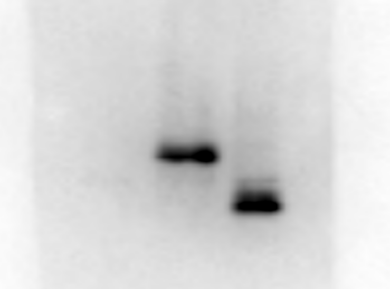


- 70 kD

- 55 kD

β-actin


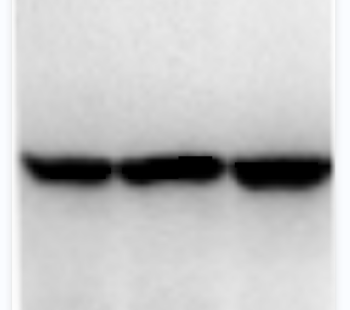


- 40 kD

**Figure 3F**

HNF4α


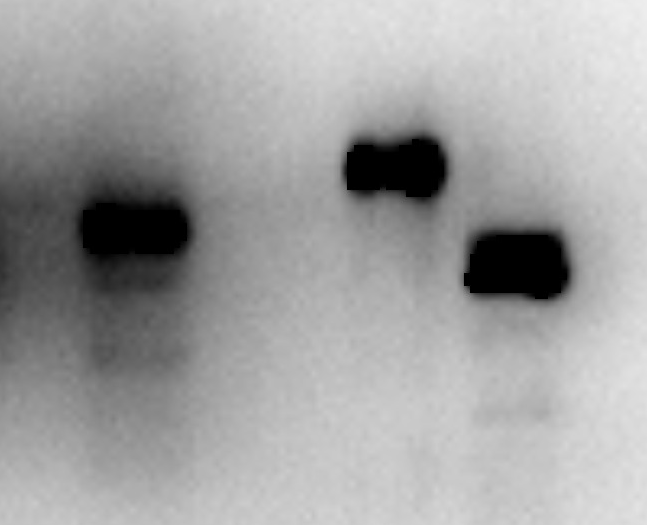


- 70 kD

- 55 kD

- 40 kD

β-actin


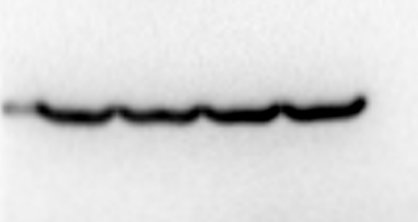


- 40 kD

**Figure 5J**

**HGC-27**

HNF4α


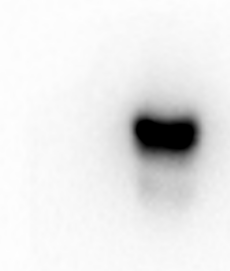


- 55 kD

HKDC1


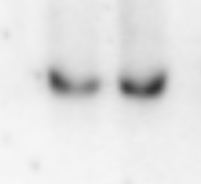


- 130 kD

- 110 kD

β-actin


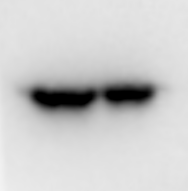


- 40 kD

**OCUM-1**

HNF4α


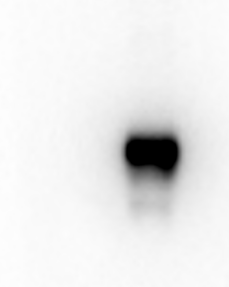


- 55 kD

HKDC1


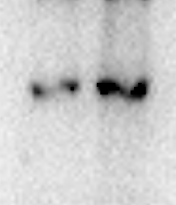


- 130 kD

- 110 kD

β-actin


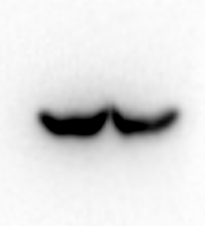


- 40 kD

**AGS**

HNF4α


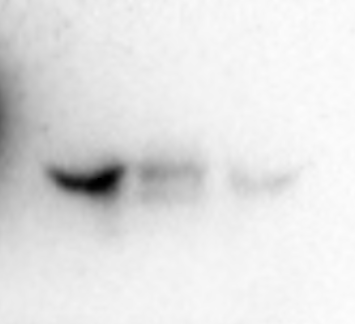


- 55 kD

HKDC1


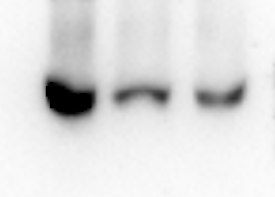


- 130 kD

- 110 kD

β-actin


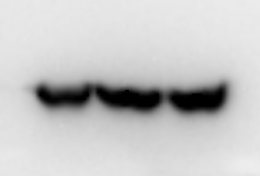


- 40 kD

**Figure 7F**


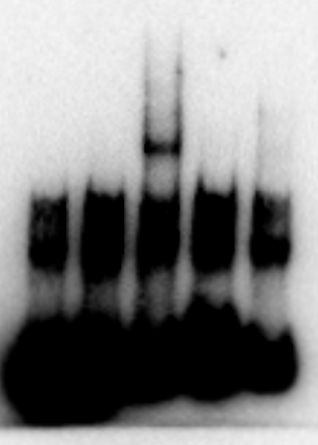

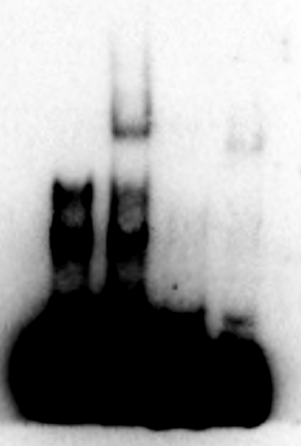


**Figure 8H**

HKDC1


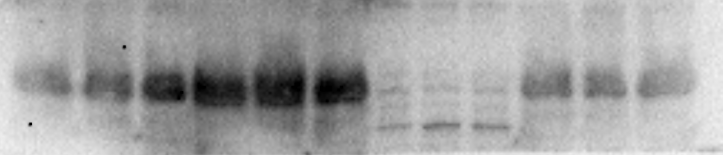


- 130 kD

- 110 kD

HNF4α


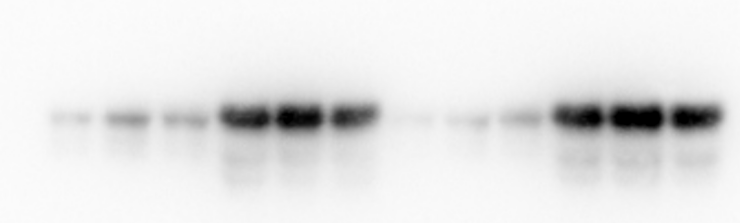


- 55 kD

β-actin


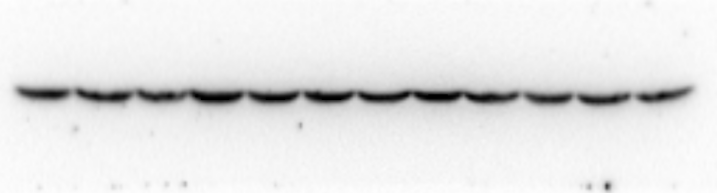


- 40 kD

**Figure S2B**

**AGS**

HNF4α


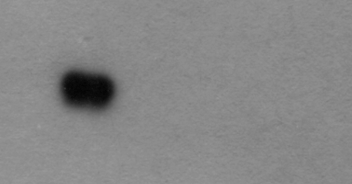


- 55 kD

β-actin


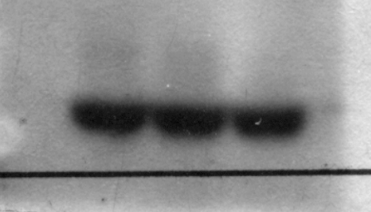


- 40 kD

**NUGC-4**

HNF4α


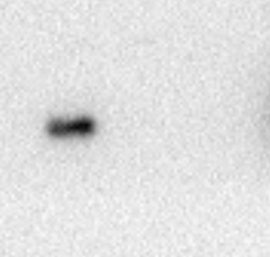


- 55 kD

β-actin


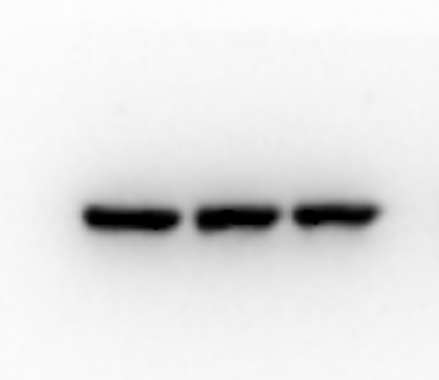


- 40 kD

**KATO-III**

HNF4α


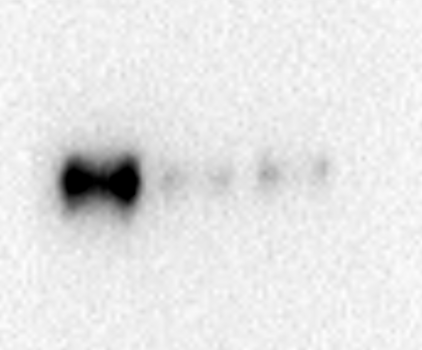


- 55 kD

β-actin


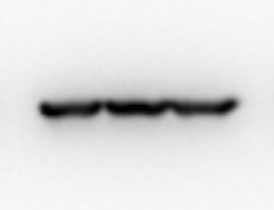


- 40 kD

**Figure S2E**

**HGC-27**

HNF4α


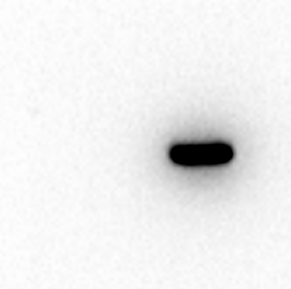


- 55 kD

β-actin


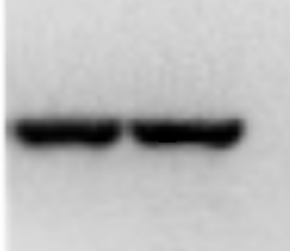


- 40 kD

**OCUM-1**

HNF4α


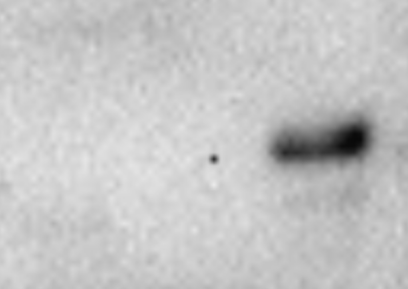


- 55 kD

β-actin


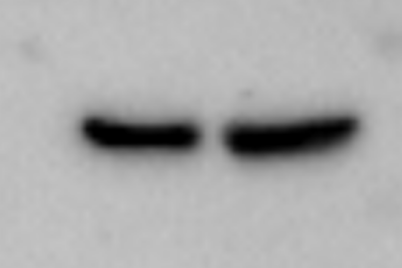


- 40 kD
